# Network structure of resource use and niche overlap within the endophytic microbiome

**DOI:** 10.1038/s41396-021-01080-z

**Published:** 2021-08-19

**Authors:** Matthew Michalska-Smith, Zewei Song, Seth A. Spawn-Lee, Zoe A. Hansen, Mitch Johnson, Georgiana May, Elizabeth T. Borer, Eric W. Seabloom, Linda L. Kinkel

**Affiliations:** 1 Department of Veterinary Population Medicine, University of Minnesota, St Paul, MN USA; 2 Department of Plant Pathology, University of Minnesota, St Paul, MN USA; 3 Department of Geography, University of Wisconsin, Madison, WI USA; 4 Center for Sustainability and the Global Environment (SAGE), University of Wisconsin, Madison, WI USA; 5 Department of Microbiology & Molecular Genetics, Michigan State University, East Lansing, MI USA; 6 Department of Horticultural Science, University of Minnesota, St Paul, MN USA; 7 Department of Ecology, Evolution and Behavior, University of Minnesota, St Paul, USA

**Keywords:** Microbial ecology, Microbiome, Community ecology, Microbial ecology

## Abstract

Endophytes often have dramatic effects on their host plants. Characterizing the relationships among members of these communities has focused on identifying the effects of single microbes on their host, but has generally overlooked interactions among the myriad microbes in natural communities as well as potential higher-order interactions. Network analyses offer a powerful means for characterizing patterns of interaction among microbial members of the phytobiome that may be crucial to mediating its assembly and function. We sampled twelve endophytic communities, comparing patterns of niche overlap between coexisting bacteria and fungi to evaluate the effect of nutrient supplementation on local and global competitive network structure. We found that, despite differences in the degree distribution, there were few significant differences in the global network structure of niche-overlap networks following persistent nutrient amendment. Likewise, we found idiosyncratic and weak evidence for higher-order interactions regardless of nutrient treatment. This work provides a first-time characterization of niche-overlap network structure in endophytic communities and serves as a framework for higher-resolution analyses of microbial interaction networks as a consequence and a cause of ecological variation in microbiome function.

## Introduction

Persistent nutrient amendment (*e.g*. of nitrogen, phosphorus, potassium, and other essential elements; hereafter “NPK”) often leads to reduced diversity [1–3] and community stability [4], but increased productivity [5]. These, in turn, influence the composition and phenotype of soil and root microbes in grassland communities [6, 7]. Increased nutrient supply rates have been correlated with reductions in soil microbial growth efficiencies and the breadth of nutrients used by soil microbes [8], and have been shown to influence both leaf nutrient composition [9–12] and plant metabolite production [13–15]. In particular, NPK amendment increases both macronutrient (i.e. N, P, and K; 9, 11), and micronutrient (e.g. Ca and Zn; 11) levels within plant leaves, as well as altering plant carbon allocation [15] and production of enzymes [14] and defensive compounds [13]. Against this backdrop of widespread direct and indirect effects of NPK amendments on plants and their associated soil communities, recent work has begun to explore the impacts of nutrient amendments on foliar endophytes; [16, 17] Kinkel unpublished).

Microbial symbionts are critical to plant health and productivity [18–23], yet the effects of individual taxa can vary from mutualistic, as in the case of nitrogen-fixing rhizobacteria and mycorrhizae, to antagonistic, as in the case of pathogenic bacteria and fungi. Moreover, host-symbiont interactions represent but a small fraction of the total, complex network that makes up the phytobiome. Substantial evidence exists that both a microbiome’s composition and its concomitant web of interactions can have dramatic Within-host Microbial Interactions and Plant Parasites: From Pairwise Interactions to the Microbiomeeffects on host plants [24–26], and that these effects can differ from those expected from summing the effects of all pairwise relationships [27–29], but see 4). Compositional changes in endophytic bacterial and fungal communities in response to NPK amendment have been shown in some cases;[17, 22, 30, 31] Kinkel unpublished). Even when the taxonomic composition is unchanged, however, functional (phenotypic) characteristics of fungal endophytic communities can shift in response to NPK amendment (16; Kinkel unpublished). In particular, resource use phenotypes among fungal, but not bacterial, communities were seen to be significantly different in leaves from nutrient-amended vs. control plots (Kinkel unpublished)). While shifts in resource use among endophytic populations in response to changes in nutrient amendments may not be surprising, the causes of these shifts and their implications for microbiome dynamics and function are unknown [21, 23, 32]. Critically, it remains to be answered whether or not the extensive effects of nutrient amendment on microbial composition and phenotype extend into the structure of the microbial interaction network.

Network analysis is an approach for analyzing systems of interconnected components and is used across a range of disciplines [33–39]. In biological systems, network analyses have proven particularly appropriate in the study of microbiomes, where hundreds of microbes can interact in complex ways [32, 39–44]. In general, network analysis seeks to answer questions about patterns of connection (are their groups of strongly interacting individuals?; are specialist interactions a subset of generalist interactions?) and influence (do some individuals have disproportionate impacts on the rest of the community?). Yet attempts to integrate network science into ecology, which involves applying methods developed idiosyncratically across multiple scientific disciplines to ecological questions, have proved challenging. In particular, ecologists have struggled to link metrics of network structure to biologically relevant variation in community composition, diversity, or stability, as well as to key environmental variables such as nutrient inputs, abiotic factors, or disturbance [39, 45].

Here, we characterize the network structure of microbial resource-niche overlap within endophytic communities in plants that have received long-term soil NPK amendments and from non-amended plants. We evaluate the roles of bacterial and fungal populations within niche-overlap networks considering local (node- or isolate-specific) measures of network structure and highlight differences between fungal and bacterial populations from nutrient-supplemented versus control leaves. Next, we determine how nutrient supplementation influences network structure, comparing global and triad-scale network structures between nutrient-amended and control plots. Finally, we investigate the possibility of higher-order interactions (HOI), in which the interaction strength or direction between two species is modified by the presence of a third species [46]. We assess the prominence of HOI by comparing each network’s observed structure to expectations based on the distribution of pairwise interactions.

Notably, we are not constructing co-occurrence networks, in contrast to the majority of studies looking at microbiome composition in plants, animals, and the environment. Co-occurrence networks are constructed by measuring the relative abundances of operational taxonomic units (OTUs) within individual samples and linking OTUs to one another if their abundances correlate across space, conditions, or time. However, strong correlations in abundance are, at best, implied interactions between isolates, providing limited insight into explicit interaction structure [39, 47]. Yet, microbes interact with one another in a variety of concrete ways. They produce antibiotics that can be used to directly inhibit their neighbors or to act as signaling compounds at lower concentrations [48]. They interact through the consumption of one another’s metabolic by-products and through the secretion of enzymes that digest carbon sources indiscriminately. Finally, they compete in the consumption of limited resources in their environment. We focus on this final type of interaction, looking at resource use phenotypes to infer competitive interactions between isolates. This work introduces a framework for the study of interaction networks among bacterial and fungal foliar endophytes in the presence or absence of an experimental treatment. These analyses provide insight into those factors that influence the structure of endophytic interactions and provide a framework for future analyses of microbial interaction networks in relation to biotic and abiotic factors.

## Materials & methods

### Data

This research utilized experimental plots at the University of Minnesota’s Cedar Creek Ecosystem Science Reserve, part of the U.S. National Science Foundation’s Long-term Ecological Research network. The experimental plots are part of the global Nutrient Network (www.nutnet.org) experiment and have received annual additions of Nitrogen, Phosphorus, and Potassium (NPK) since 2007, as well as a micronutrient mix applied once at the start of the experiment [17, 49, 50].

We focused on endophytic microbes associated with the tallgrass prairie species *Andropogon gerardii* (big bluestem). In early September 2015, a single healthy mature leaf was collected from each of 6 different plants within each treatment (nutrient-amended soil, non-amended). Plants were stored on ice during transport to the lab, and subsequently surface-sterilized by immersing individual leaves for 1 min each in: sterile deionized water, 75 % ethanol, 0.4125 % sodium hypochlorite (bleach solution), 75 % ethanol, and sterile deionized water. Leaves were immediately sectioned into 3 pieces of approximately equal size using a flamed scalpel. One section of each leaf was randomly selected for further study here.

Individual leaf sections were placed in 10 ml PBS buffer and macerated using a Fisher Scientific™ Handheld Homogenizer (FSH 125) for 3 min. The resulting macerate and three dilutions (of 10^*−*2^, 10^*−*4^, and 10^*−*6^) were each plated onto multiple nutrient media, including: malt extract agar, water agar, starch-casein agar, and pentachloronitrobenzene peptone agar. Plates were incubated at either room temperature or 28 °C and were checked periodically for newly emerging isolates. Microbes were randomly selected from each medium for further study, purified through successive transfers, and stored either in 20% glycerol stock suspensions (bacteria, −80 °C) or on agar slants (fungi, 4 °C).

From the resulting collection of over 800 microbial isolates (spanning 35 genera: 17 bacteria and 18 fungi; Tables S1 and S2), we randomly selected up to 10 bacterial and 10 fungal isolates from each leaf for further investigation. We refer to these collections of co-occurring microbes as “communities” explicitly assuming that each collection was sampled from a sympatric community existing within a host leaf. For each microbial isolate, we evaluated resource use on 95 carbon substrates using Biolog SF-P2 plates (Biolog, Hayward, CA), as described previously [6, 51–55].

### Niche overlap calculation

Growth efficiencies on each nutrient, quantified by optical densities, were used to formulate a resource niche for each isolate, and relative niche-overlap was calculated for each ordered pairwise combination of isolates. Specifically, we calculate the pairwise overlap

1 $$\omega _{i \to j,n} = \min \left( {\frac{{g_{i,n}}}{{g_{j,n}}},} \right)$$

for each of the *m*(*m −* 1) ordered pairs of isolates *i* and *j* (where *m* is the number of unique isolates) and for each substrate *n* for which both isolates show non-zero growth. *g*_*i,n*_ indicates the optical density of isolate *i* after 72 h of growth on nutrient source *n*. This value can be thought of as the fraction of isolate *j*’s growth on substrate *n* that is matched by isolate *i*. We average these values across substrates to get a single value for each ordered pair of isolates:

$$\bar \omega _{i \to j} = \frac{1}{{95}}\mathop {\sum }\limits_{n = 1}^{95} \omega _{{i \to j}, n}.$$

*N.b*. this approach explicitly treats all 95 substrates as equally important in defining an isolate’s resource niche. These mean, relative, pairwise niche overlap values (hereafter NO) were taken as a proxy for the strength of competitive interaction of each isolate against each other isolate and combined to form directed niche-overlap networks for each of the communities. With the exception of one of the control leaf sections for which we were unable to collect ten bacterial isolates, each of these networks consisted of twenty nodes (representing ten fungal and ten bacterial isolates). In the case of the exception, only five bacterial isolates were collected, leading to a smaller network consisting of fifteen nodes (ten fungal and five bacterial). Previous work has shown that niche overlap metrics calculated using such data are significantly correlated with antagonistic phenotypes among coexisting microbes, supporting their use as a metric of interaction [54, 56]. Sensitivity to this formulation of niche overlap is explored in the Supplementary Information (Fig. S1 and S2 and Tables S3 to S2).

### Creating a directed binary network

The above process produces a weighted (*i.e*. non-binary) niche-overlap value for each pair of isolates. While such an approach has the potential to offer additional nuance to our understanding of the overall network structure, in most cases we found the results did not differ qualitatively from those for binary representations unless otherwise noted. Thus, while we report results for the weighted networks in the Supplementary Information (Fig. S3 to S5 and Tables S7 to S12), we focus on binary networks in the main text. We use a threshold cutoff for NO in order to create a binary interaction network. Here, we define a significant resource-competitive interaction as a NO of greater than or equal to 75%. The sensitivity of results to cutoff value is explored in the Supplementary Information (Fig. S6 and S7 and Tables S13 to S18). Explicitly, for each pair of isolates *i* and *j*, if Eq. (1) yields a value greater than 0.75, we say that *i* has a significant niche overlap on *j*. We signify this in the network by drawing an arrow: *i → j*. Importantly, just because *i* significantly overlaps *j* does not mean the reverse is true, *i.e*., in general, ${\bar{\omega}}_{i\rightarrow j}\;\ne\;{\bar{\omega}}_{j\rightarrow i}$). Binary networks are plotted using a Fruchterman-Reingold algorithm in which nodes are placed in space according to a balance of interactions pulling nodes together and an underlying inter-node repulsion, resulting in groups of connected nodes being placed more closely together than more disconnected ones. This visual grouping is evaluated statistically using a spin glass algorithm [57] to identify community structure in the network and comparing community membership to isolate kingdom using a *χ*^2^-test.

### Network structure metrics

One of the most fundamental properties of network structure is the degree distribution, i.e. in how many competitive interactions does each isolate participate? Because our networks are directed, we are interested both in an isolate’s indegree (number of other isolates whose resource niche significantly overlaps a focal isolate’s resource niche) and its outdegree (number of other isolates for which the focal isolate’s resource niche significantly overlaps their resource niches). To characterize the shape of the degree distribution, we measure several summary statistics, including the mean, standard deviation, and skewness of the in- and out-degree distributions. We also consider a combined degree measure: the proportion of total degree that is inbound. We calculate mean, standard deviation, and skewness for this distribution across nodes as well.

In network theory, centrality measures have been developed to quantify the importance of nodes within a network, and the many different types of centrality differ in how they define importance. Degree centrality measures the total number of incoming and outgoing links from a node. Thus, isolates that have many and strong interactions will have a higher degree of centrality. Closeness centrality, on the other hand, looks at the wider pattern of interactions, asking how many links separate a given node from all other nodes in the network. A node with high closeness centrality will have short paths to other nodes within the network. In more tangible terms, this can be interpreted as an isolate’s influence on other isolates in the community (and vice versa) being more proximate. We calculate these two centrality measures for each isolate.

Finally, we calculate a suite of metrics that measure key characters about the potential competitive links within the network. These include: (i) clustering coefficient; [58] (ii) intransitivity, which has been suggested to be key for maintaining diversity through the promotion of coexistence (Allesina and Levine [59], Maynard et al. [60, 61], but see Gallien et al. [62]; and (iii) triad counts, i.e. the number of each unique three-node subgraph (“triad”) present in a network [63, 64]. Triad counts have received attention recently as intermediate-scale structures and potential “building blocks” of whole-network structures. Formal definitions of each of these metrics can be found in the Supplementary Information.

For each of the metrics above, we compared across isolate kingdoms (bacteria or fungi) or nutrient treatments (NPK amended or control) using Welch’s *t*-Tests.

### Differences of observed network structure from pairwise expectations

While the biological effects of higher-order interactions (HOI) have rarely been quantified in empirical systems [27, 65–67], one way in which the presence or absence of such effects can be evaluated is in deviation from predictions based on pairwise measurements [46, 68]. In a network context, we can identify higher-order interactions when aspects of the network structure (e.g. the degree of clustering or the counts of particular triads) deviate from what we would expect in a network constrained solely by the number and positioning of pairwise interactions. To this end, we compare each structural metric of the empirically-assembled networks from each host leaf to the distribution of that metric generated from randomizations that preserve the in- and outdegree distributions of each node (microbial isolate) in the network, but randomize the connections between isolates (i.e. a configuration model *sensu* [69]).

## Results

**Fig. 1:**
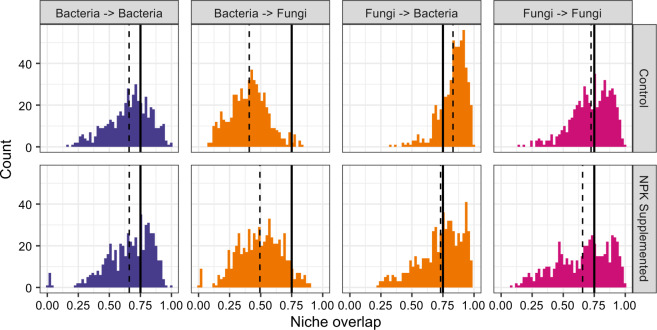
Distributions of niche overlap calculated by averaging the nutrient-wise overlaps between each pair of co-occurring isolates. We divide these values according to treatment and interaction focal/partner kingdom. The solid black lines in each panel indicate the 0.75 thresholds utilized to formulate binary interaction networks from these values, while the dashed lines indicate the mean of each distribution. All distributions are significantly different from one another in both mean and shape with the exception of the Bacteria → Bacteria distributions across treatments (Table S19).

Co-occurring (within the same leaf segment) endophytic bacteria and fungi exhibited a wide range of mean pairwise niche overlap (NO) values. Distributions of NO differed between organisms from the same vs. different kingdoms and in the presence vs. absence of long-term NPK amendment (Fig. 1). While the percent NO between any two isolates can range from 0 to 1, we observed highly non-uniform distributions of NO, and distributions varied significantly depending on nutrient treatment as well as kingdom of focal and partner isolate (Fig. 1 and Table S19). For fungi competing against bacteria or other fungi, and for bacteria competing against other bacteria, we observed a high degree of NO among most pairs of co-occurring endophytes. In contrast, bacterial NO on co-occurring fungal isolates tends to be smaller; this is partially a consequence of the larger average niche widths of fungi compared to bacteria (Welch’s *t*-Test *p* < 0.001; Kinkel unpublished). Collectively, the distribution of NO values highlights the diversity of interaction patterns among microbes and draws attention to differences between kingdoms.

### Analysis of the roles of bacteria and fungi in endophytic networks

**Fig. 2:**
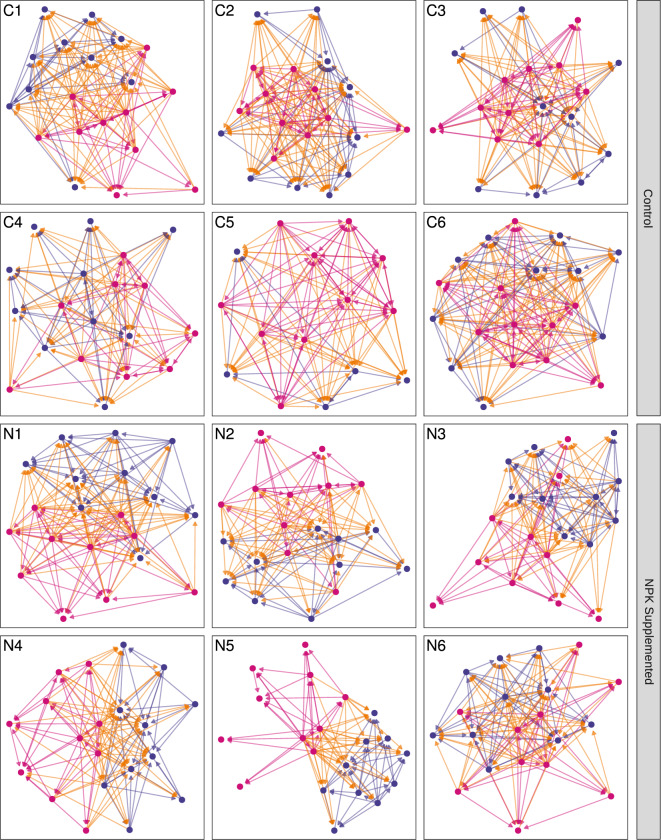
Network diagrams of each of the twelve niche-overlap networks (one for each leaf, numbered 1–6 for each treatment, control “C” and NPK Supplemented “N”) generated by measuring resource use across 95 carbine substrates using Biolog MicroPlates. Blue nodes signify bacterial isolates and pink nodes signify fungi. Links between nodes of the same kingdom share the color of the nodes being connected, links that connect across kingdoms are colored orange. Nodes are arranged according to the Fruchterman-Reingold algorithm; any spatial patterns are emergent properties of overall networks structure, though care should be taken in interpreting node positioning in the absence of statistical analysis. All plotted networks consist of twenty nodes (ten bacterial and ten fungal), with the exception of control leaf five (for which only five bacterial isolates were obtained), and control leaf four (“C4”; for which one fungal isolate was disconnected from the giant component following thresholding of the niche overlap). This disconnected node was omitted to improve visual clarity, but included in all other analyses.

Using the binary interaction data (NO greater than 75%), we constructed networks for each community, detailing all pairwise, directed interactions between isolate pairs (Fig. 2). When plotted, a visual grouping of nodes according to the kingdom is apparent. This “community structure” illustrates that isolates have greater NO on average with members of the same kingdom than with members of the other kingdom. However, when investigated statistically, only a few of the NPK-supplemented networks show significant grouping of isolates by kingdom (Table S20; [57, 70]). Note that when weighted networks are considered (i.e. using the raw NO values as interaction strengths), the strength of intra-kingdom grouping is stronger in the Control treatment, relative to those undergoing NPK supplementation (Table S20).

Considering each endophytic community independently, we found differences in both degree and closeness centrality between bacterial and fungal isolates (Table 1). While results were not consistent across all networks, bacteria and fungi differed most frequently within the control treatment and when considering the degree of NO. Differences between fungi and bacteria in degree or closeness centrality were rarely significant in nutrient-amended leaves (Table 1). In control leaves, patterns of network structure were dominated by fungal isolates overlapping a (usually bacterial) co-occurring isolate’s resource niche. Thus, bacteria have higher indegree on average, while fungi have higher outdegree. Moreover, bacteria have higher closeness centrality when inbound links are considered (they tend to have their resource niches overlapped by others), while fungi have higher closeness centrality when considering outbound links (they tend to be more dominant resource users). These differences between bacteria and fungi were attenuated in the NPK-treated communities (fewer significant differences in local structure between kingdoms), consistent with an “equalizing” effect of nutrients on bacterial-fungal resource competitive interactions in the presence of NPK (Table 1). Finally, fungal isolates were less clustered than bacterial isolates (i.e. when a bacterium interacts with two other isolates, those two isolates tend to more often interact with each other, forming dense aggregates of interacting microbes, whereas fungal interaction partners are less likely to interact with one another; 58). This is due in part because the bacterial interaction partners of fungi tend to have smaller niche widths, limiting their ability to interact with the other interaction partners of a focal fungal isolate.

**Table 1:** Welch’s Two-Sample *t*-Test comparing clustering and two measures of centrality (degree and closeness) between bacterial and fungal isolates within each network.

Each column represents an individual leaf, ordered as in Fig. 2 (C1-C6 and N1-N6). The *p* values have been corrected for multiple comparisons [80]. When differences are significant, boxes are colored according to the isolated kingdom with the larger value for each metric ( for bacteria and  for fungi). The intensity of the color indicates the level of significance: /, /, and / shades signifying *p* values < 0.001, < 0.01, and < 0.05, respectively. Empty boxes signify *p* values > 0.05. Formal definitions of each metric can be found in the Supplementary Information.

**Fig. 3:**
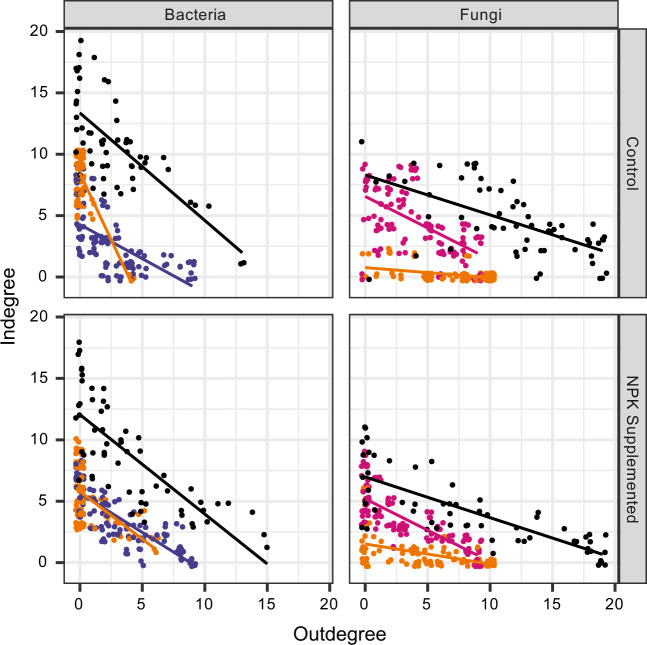
In- by outdegree for each isolate according to isolate kingdom and host plant treatment. Panels are divided according to nutrient treatment (rows) and focal isolate kingdom (columns). Colored points indicate the kingdom of the focal (for indegree) or partner (for outdegree) isolate: orange indicates interactions between kingdoms, while within kingdom links are colored according to the kingdom, using the same colors as in Fig. 2 (pink for fungi, blue for bacteria). Black points indicate values for each isolate interacting with all others (bacteria and fungi). Thus, there are three points for each isolate in each panel: one for their interactions with other members of their same kingdom (blue or pink for bacteria or fungi, respectively), one for interactions with the alternate kingdom (orange), and one for their total number of interactions (black). Links are assigned when an isolate has greater than 75% niche overlap with another isolate. Points do not fall exactly on integers due to slight jittering to improve readability. Lines indicate best-fitting linear models for each subset of the data. All slopes are significantly different from 0; Table 2).

Among all bacterial and fungal isolates, there was a negative relationship between in- and outdegree: isolates whose niches strongly overlapped their neighbors generally had fewer cases where their neighbors’ niches strongly overlapped their own (Table 2). The strength of the imbalance between in- and outdegree is captured by the slope of the relationship between in- and outdegree (Fig. 3). This slope was steeper for fungi than for bacteria (Table 2 and S3; slopes ranging from −2.16 to −0.08). Slopes were not related to hosting plant nutrient treatment, but were consistently more steeply negative when the focal isolate was a bacterium or when the partner isolate was a fungus (Table S21). Interestingly, the effect of taxonomy on the relationship between in- and outdegree was largely constrained to the kingdom, with finer classifications having a minimal contribution to explaining the variation (Table S22). Additional taxonomic considerations are addressed in the Supplementary Information (Fig. S8 to S10).

**Table 2:** Linear model results for indegree by outdegree in each sympatric network, differentiated according to focal and partner isolates’ Kingdom (each unique combination of color and panel in Fig. 3).

This analysis utilizes an average pairwise measure of niche overlap and binary interaction strengths. An analysis of variance in these slopes is presented in Table S21.

When considering nutrient treatment, interactions where the partner isolate was a bacterium (*i.e*. Bacteria *→* Bacteria and Fungi *→* Bacteria) tended to be more steeply negative in leaves undergoing nutrient amendment, whereas interactions with fungal partners (i.e. Bacteria *→* Fungi and Fungi *→* Fungi) were less steeply negative (Table S21). The steepness of the relationship between in- and outdegree can be construed as a group measure of competitive ability. For a group to have a steep slope in Fig. 3, the component isolates must have high indegree relative to their outdegree; *i.e*. they must be weaker competitors on average. In contrast, groups with shallower slopes have component isolates with higher outdegree relative to their indegree; *i.e*. they tend to dominate in their interactions with other isolates. Importantly, this explanation does not take into account the potential life-history strategy of being a specialist on an uncommon resource: such isolates would have low in- and outdegree; *i.e*. they would have few interactions in general. Additionally, all inference of interaction strength from our calculations of niche overlap will depend in part upon the complement of resources in situ.

### Metrics of network structure

There were few significant differences in common measures of network structure across treatments and none that were robust to correction for multiple hypothesis testing (Table 3, S23, and S24). That is, despite differences in the degree distributions, and differences in relationships between in- and outdegree in nutrient-amended and control leaves, we did not see differences across nutrient treatments in network metrics including whole-network clustering, intransitivity, and summary statistics of the degree distribution. Likewise, there were few significant differences in triad counts between nutrient treatments (Table 4; but see Supplementary Information).

**Table 3:** Welch’s two-sample *t*-Test comparisons of whole-network-scale metrics across treatments.

Boxes are colored according to the treatment with the larger value for each metric,  for control, and  for NPK. Color intensity indicates the level of significance, with / and / signifying *p* values < 0:01 and < 0:05, respectively. Empty boxes signify *p* values > 0:05. *p* values are uncorrected for multiple hypothesis testing and applying such a correction makes all differences non-significant. We omit the outlying control network with fewer than twenty isolates; inclusion of this community does not change these results qualitatively. Formal definitions of each metric can be found in the Supplementary Information.

**Table 4:** Welch’s two-sample *t*-Test comparisons of network triad counts across treatments.

Boxes are colored according to the treatment with the larger value for each metric,  for control, and  for NPK (none present). Empty boxes signify *p* values > 0.05. × indicates cases where both treatments had no instances of that particular triad. The single significant difference has a *p* value of 0.028. The *p* values are uncorrected for multiple hypothesis testing and applying such a correction removes all significant differences. We omit the outlying control network with fewer than twenty isolates; inclusion of this community causes one other relationship to become significant: Triad 2 becomes significantly higher in NPK relative to Control plants.

### Is there higher-order structure in endophytic microbial community interaction networks?

Looking at a wide range of network metrics, we probed for the presence of higher-order interactions (HOI) by comparing the empirically measured values for these metrics to expectations based solely on each network’s pairwise interaction structure (Table 5). We found that HOI was uncommon: most empirical networks’ structures were indistinguishable from their randomizations based on pairwise interactions. Yet, there were some idiosyncratic discrepancies from expectation, concentrated in (i) particular networks, e.g. Control network 4 and NPK networks 1 and 5; and (ii) particular metrics, e.g. triads with two or fewer interactions. This finding is consistent with some prior work suggesting that only a subset of microbes participate in HOI [27, 67].

**Table 5:**
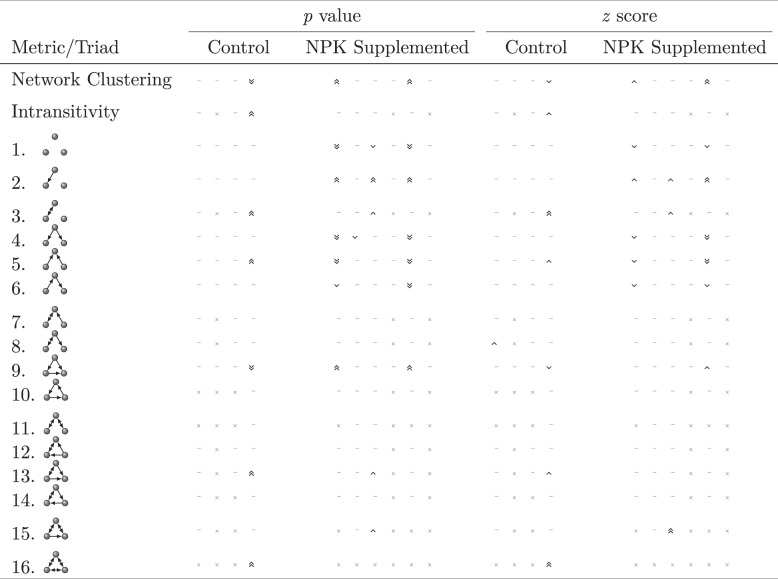
Summary test for higher-order interactions in endophytic microbial networks.

Empirical *p* values (left) and *z* scores (right) for network structure metric comparisons between empirical and randomly-rewired networks. Each column represents an individual leaf, ordered as in Fig. 2 (C1-C4 and N1-N6). Values less than 0.05 (−2) signify that the empirical value is significantly smaller than expected, and are represented by  ( for values less than 0.01 (−4)). Likewise, values greater than 0.95 [2] signify that the empirical value is larger than expected and are represented by  ( for values greater than 0.99 [4]). Dashes (─) signify values between 0.05 and 0.95 (−2 and 2; i.e. non-significant differences) and ╳ indicates cases where all randomizations resulted in the same value for this metric/community combination. Two of the control leaves (C5 and C6) were omitted from this analysis because 1000 randomizations of these networks yielded only 1 and 4 unique network configurations, respectively.

In addition to calculating empirical *p* values (which indicate *whether or not* an empirical value differs from expectations), we also calculated *z* scores, which are a measure of the *magnitude* of any differences (Table 5). We found fewer substantial deviations (empirical values more than two standard deviations from the mean of the distribution measured for the randomized networks) than we saw significant differences in the *p* values, signifying that some of the statistically significant deviations may be less likely to represent biologically meaningful differences. In all cases, the direction of the effect was consistent across networks within the same treatment.

Taken together, our results suggest that HOI, when present, are highly localized, both within particular networks, and within particular structures in a given network; yet, the structures of the endophytic networks analyzed here are largely constrained by their pairwise interactions. This is consistent with previous results that have suggested that, at least in small communities, the effects of HOI are often overshadowed by the effects of pairwise interactions [71].

## Discussion

While NPK amendments have been shown to have diverse effects on microbial communities, it had not yet been addressed whether these effects percolate into the structure of the interaction networks of naturally-assembled microbial communities. We used network analyses to characterize the structure of species interactions within endophytic microbiomes under distinct nutrient regimes. We found that the addition of NPK to hosts plants altered networks so that the structure of bacterial and fungal interactions became more similar to one another, suggesting that the role of cross-kingdom interactions in community-wide dynamics is altered significantly with plant nutrient conditions. Second, we found that these changes were due to changes in the degree distribution among isolates, especially reflecting changes in fungal niche overlap, but not to changes in global network structure. Finally, there was limited evidence for higher-order interactions (HOI) in endophytic networks, though this varied for communities from different leaves.

### Nutrient amendments reduce inter-kingdom differences

When perennial host plants are exposed to long-term NPK amendments, the interaction network patterns among endophytic fungi become more similar to those of co-occurring bacteria. This reflects a reduction in fungal niche overlap against fungi and bacteria, as well as an increase in the overlap of bacteria on fungi (Fig. 1). This is illustrated clearly in Fig. 3 by the increase in Bacteria*→*Fungi interactions in NPK-amended treatments relative to control treatments. Moreover, in contrast to control leaves, when considering clustering and centrality there are almost no significant differences between fungi and bacteria in NPK-amended communities (Table 1), consistent with a loss in fungal competitive advantage.

### Nutrient treatments alter degree distribution, but not global network structure

The significant loss in fungal competitive advantage, and corresponding differences in degree distribution with NPK amendment, did not translate into significant differences in global network structure between treatments. Though endophytic communities from non-amended leaves had greater clustering and connectance, consistent with a greater role of species interactions in community dynamics, these differences were not statistically significant (Table 1). This may suggest a form of functional replacement at the level of network structure, whereby nodes with different degree signatures nonetheless fill similar roles as components of larger structures within the network. This could reflect some form of higher-order selection, for instance on stabilizing network structures [72], that constrains endophytic network structure. Indeed, the ranking of relative abundance of triads 7–10 in our networks maps exactly on to a ranking of relative stability noted by Borrelli et al. [72]. Triads 9 and 13 were also found to be common in the soil communities analyzed by Schlatter et al. [63], and were usually, though not always more represented than other triads with the same number of interactions.

Alternatively, the lack of differences in larger network structures could result from limitations to capturing network structure through community sub-samples. Recent work on animal movement networks has suggested that, while local network structures such as degree are relatively easy to recover without comprehensive sampling, more complicated network structures are nearly impossible to measure accurately without infeasible sampling coverage [73, 74]. In the case of endophytic microbiomes, where there are potentially up to hundreds of microbes interacting in close proximity, a sub-sample of twenty microbes may simply be too few to detect the differences present in the wider network structure, despite capturing differences in-degree distribution. Finally, it could be that there is too much variation among communities within each treatment to successfully distinguish differences in network structure among treatments. Further study of naturally-assembled endophytic microbial community interaction networks across a range of conditions and with larger sample sizes is needed to resolve these questions.

### Deviations from expected resource-competitive network structure are rare

There is significant interest in HOI, in which interactions between a collection of organisms are altered by the presence of other organisms, both as a means of making ecological models more realistic and for understanding their potential role as a stabilizing force in large, complex communities [27, 75]. One of the common approaches for identifying HOI in interaction networks is to compare an empirical network structure to an ensemble of random networks generated by rewiring the empirical network while conserving its pairwise structure (i.e. degree distribution). Differences between the empirical and randomly-rewired networks can be viewed as evidence for HOI. While recent research has suggested that HOI are both common [27, 76, 77] and important to community dynamics [67, 75], in this work we found little evidence for either claim. Overall, there were few significant differences between observed and predicted network structures, and *z* scores suggested that many significant differences from the rewired networks were small.

One limitation to our analysis of HOI is the fact that several of the endophytic networks had relatively few possible configurations with the same degree distribution. Most dramatically, two of the communities’ structures were nearly completely dictated by their degree distribution; out of at least 1000 attempted randomizations, fewer than five unique network configurations were found. Even among networks that had more unique randomizations, four additional networks yielded fewer than twenty unique combinations of all metrics collected. In only one case (leaf C4) did we find 1000 unique randomized networks in our sampling. In other words, for most of these communities there are very few possible distinct network configurations with the empirical degree distributions. This is not a limitation resulting from network size, but rather from these particular networks’ highly nested structure [78]. Further research is needed to distinguish whether this nestedness is merely a consequence of experimental sampling design, or if it is representative of endophytic resource-competitive networks more generally.

### The importance of empirical systems

The study of microbial community assembly and structure has been driven forward on two major fronts. First, there is an enormous body of data on the relative abundance of microbial populations across communities in natural habitats, allowing the analysis of dynamics and construction of co-occurrence networks across time and space. These data often have a sampling intensity and complexity that reflects the real world, but introduce assumptions about potential interactions between species that are not readily verifiable [39, 47]. However, empirically measure microbial interactions typically requires more work or resource-intensive approaches, such as culturing microbes in isolation or in small, synthetic communities [79], or inferring resource use from metatranscriptomics or radioactive labelling.

Here, we provide a third path toward understanding microbiome assembly and function, complementary to these first two. Isolation and characterization of interaction structures for subsampled communities that have resulted from natural community assembly processes have the potential to yield essential insights into the organization of microbial interactions in situ. Notably, naturally occurring microbial communities are generally much larger than sampled here, which inevitably influenced our ability to detect the full complexity of endophytic competitive interaction network structure. For instance, we find a complete turnover of isolates between communities, something which may not be expected with comprehensive sampling. As a consequence, while looking for generalizable patterns across communities within a treatment, these efforts were potentially hampered by the possibility of exaggerated inter-community variation. Likewise, we rely upon culturability to identify microbial isolates, introducing a potential bias to our sampling. Nevertheless, our approach provides an important step forward in strategies for studying microbial community interaction networks in natural communities as an essential complement to the outstanding work being conducted at the bench and chalkboard.

## Conclusion

Endophytes are a critical component of healthy, productive plant communities, and have important and sometimes dramatic effects on their hosts. While much research has focused on interactions among isolated species or within small, synthetic communities, this work aims to provide a framework for enhanced understanding of the structure of naturally-assembled endophytic communities and their network of species interactions. We focused on resource use and niche overlap, one important means by which microbes have the potential to interact with one another within host plants. We considered network structures for communities in NPK-amended and control leaves in three ways: (i) the network degree distribution; (ii) the global network structure; and (iii) the potential for HOI by contrasting network structure to an expectation based on randomization.

We found widespread differences between microbial kingdoms and nutrient treatments in the degree distribution of endophytic competitive in- and outdegree, in particular noting that nutrient supplementation reduced the competitive advantage of fungi over bacteria. These differences did not, however, propagate into the global network structure. We found remarkably few differences in global network structure across treatments. Finally, we found limited evidence for higher-order interactions, with significant interactions concentrated in select communities and metrics. Collectively, these findings suggest that nutrient amendments to ecosystems can significantly impact microbial interactions within endophytic communities, but that their impact on global network structure is muted. This work provides a foundation for further investigations into the nature and relevance of microbial interaction structure for community assembly and function. Further research is needed to provide higher-resolution analyses of naturally-assembled microbial communities to identify relationships between microbial interaction network structures as a consequence and cause of ecological variation, as well as their potential role in ecosystem management and conservation.

## Supplementary information

Supplementary Information

## References

[1] Borer ET, Seabloom EW, Mitchell CE, Cronin JP (2014). Multiple nutrients and herbivores interact to govern diversity, productivity, composition, and infection in a successional grassland. Oikos.

[2] Isbell F, Reich PB, Tilman D, Hobbie SE, Polasky S, Binder S (2013). Nutrient enrichment, biodiversity loss, and consequent declines in ecosystem productivity. Proc Natl Acad Sci.

[3] Robinson RJ, Fraaije BA, Clark IM, Jackson RW, Hirsch PR, Mauchline TH (2016). Endophytic bacterial community composition in wheat (*Triticum aestivum*) is determined by plant tissue type developmental stage and soil nutrient availability. Plant Soil.

[4] Ratzke C, Barrere J, Gore J (2020). Strength of species interactions determines biodiversity and stability in microbial communities. Nat Ecol Evol.

[5] Lambers JHR, Harpole WS, Tilman D, Knops J, Reich PB (2004). Mechanisms responsible for the positive diversity–productivity relationship in minnesota grasslands. Ecol Lett.

[6] Essarioui A, LeBlanc N, Kistler HC, Kinkel LL (2017). Plant community richness mediates inhibitory interactions and resource competition between *Streptomyces* and fusarium populations in the rhizosphere. Micro Ecol.

[7] Pan Y, Cassman N, de Hollander M, Mendes LW, Korevaar H, Geerts RH (2014). Impact of long-term n, p, k, and npk fertilization on the composition and potential functions of the bacterial community in grassland soil. FEMS Microbiol Ecol.

[8] Schlatter DC, DavelosBaines AL, Xiao K, Kinkel LL (2013). Resource use of soilborne *Streptomyces* varies with location phylogeny, and nitrogen amendment. Micro Ecol.

[9] Firn J, McGree JM, Harvey E, Flores-Moreno H, Schütz M, Buckley YM (2019). Leaf nutrients, not specific leaf area, are consistent indicators of elevated nutrient inputs. Nat Ecol Evol.

[10] Anderson TM, Griffith DM, Grace JB, Lind EM, Adler PB, Biederman LA (2018). Herbivory and eutrophication mediate grassland plant nutrient responses across a global climatic gradient. Ecol.

[11] Bernstein N, Gorelick J, Zerahia R, Koch S (2019). Impact of n, p, k, and humic acid supplementation on the chemical profile of medical cannabis (*Cannabis sativa L*.). Front Plant Sci.

[12] Tangolar S, Tangolar S, Torun AA, Ada M, Göçmez S (2020). Influence of supplementation of vineyard soil with organic substances on nutritional status, yield and quality of ‘black magic’ grape (*Vitis vinifera L*.) and soil microbiological and biochemical characteristics. OENO One.

[13] De Long JR, Sundqvist MK, Gundale MJ, Giesler R, Wardle DA (2016). Effects of elevation and nitrogen and phosphorus fertilization on plant defence compounds in subarctic tundra heath vegetation. Funct Ecol.

[14] Dietrich R, Ploss K, Heil M (2004). Constitutive and induced resistance to pathogens in *Arabidopsis thaliana* depends on nitrogen supply. Plant Cell Environ.

[15] Bryant JP, Chapin III FS, Klein DR. Carbon/nutrient balance of boreal plants in relation to vertebrate herbivory. Oikos. 1983;40:357–68.

[16] Kinkel LL, Otto-Hanson LK, Otto-Hansen Z, Johnson M, Spawn S, Song Z (2018). Foliar endophytic microbiome composition and functional capacities vary with soil nutrient inputs. Phytopathol.

[17] Seabloom EW, Condon B, Kinkel L, Komatsu KJ, Lumibao CY, May G (2019). Effects of nutrient supply, herbivory, and host community on fungal endophyte diversity. Ecol.

[18] Vandenkoornhuyse P, Quaiser A, Duhamel M, Le Van A, Dufresne A (2015). The importance of the microbiome of the plant holobiont. N. Phytol.

[19] Stulberg E, Fravel D, Proctor LM, Murray DM, LoTempio J, Chrisey L (2016). An assessment of US microbiome research. Nat Microbiol.

[20] Hanson BM, Weinstock GM (2016). The importance of the microbiome in epidemiologic research. Ann Epidemiol.

[21] Bell TH, Hockett KL, Alcalá-Briseño RI, Barbercheck M, Beattie GA, Bruns MA (2019). Manipulating wild and tamed phytobiomes: Challenges and opportunities. Phytobiomes J.

[22] Henning JA, Kinkel L, May G, Lumibao CY, Seabloom EW, Borer ET (2021). Plant diversity and litter accumulation mediate the loss of foliar endophyte fungal richness following nutrient addition. Ecol.

[23] Vacher C, Hampe A, Porté AJ, Sauer U, Compant S, Morris CE (2016). The phyllosphere: microbial jungle at the plant–climate interface. Annu Rev Ecol Evol Syst.

[24] Berendsen RL, Pieterse CM, Bakker PA (2012). The rhizosphere microbiome and plant health. Trends Plant Sci.

[25] Turner TR, James EK, Poole PS (2013). The plant microbiome. Genome Biol.

[26] Trivedi P, Leach JE, Tringe SG, Sa T, Singh BK (2020). Plant–microbiome interactions: from community assembly to plant health. Nat Rev Microbiol.

[27] Sanchez-Gorostiaga A, Bajić D, Osborne ML, Poyatos JF, Sanchez A (2019). High-order interactions distort the functional landscape of microbial consortia. PLOS Biol.

[28] Gould AL, Zhang V, Lamberti L, Jones EW, Obadia B, Korasidis N (2018). Microbiome interactions shape host fitness. Proc Natl Acad Sci.

[29] O’Keeffe KR. Within-host Microbial Interactions and Plant Parasites: From Pairwise Interactions to the Microbiome. PhD thesis, The University of North Carolina at Chapel Hill, 2019.

[30] Wemheuer F, Kaiser K, Karlovsky P, Daniel R, Vidal S, Wemheuer B (2017). Bacterial endophyte communities of three agricultural important grass species differ in their response towards management regimes. Sci Rep..

[31] Wemheuer B, Thomas T, Wemheuer F (2019). Fungal endophyte communities of three agricultural important grass species differ in their response towards management regimes. Microorg.

[32] Layeghifard M, Hwang DM, Guttman DS (2017). Disentangling interactions in the microbiome: a network perspective. Trends Microbiol.

[33] Barabási Albert-László (2016). Network Science.

[34] Scott J (1988). Social network analysis. Sociol.

[35] Borgatti SP, Mehra A, Brass DJ, Labianca G (2009). Network analysis in the social sciences. Science.

[36] Nelson GD, Rae A (2016). An economic geography of the United States: from commutes to megaregions. PLOS ONE.

[37] Danon L, Ford AP, House T, Jewell CP, Keeling MJ, Roberts GO, et al. Networks and the epidemiology of infectious disease. Interdiscip Perspectives on Infect Dis. 2011. 10.1155/2011/284909 PMC3062985 21437001

[38] Expert P, Evans TS, Blondel VD, Lambiotte R (2011). Uncovering space-independent communities in spatial networks. Proc Natl Acad Sci.

[39] Röttjers L, Faust K (2018). From hairballs to hypotheses—biological insights from microbial networks. FEMS Microbiol Rev.

[40] Naqvi A, Rangwala H, Keshavarzian A, Gillevet P (2010). Network-based modeling of the human gut microbiome. Chem Biodivers.

[41] Coyte KZ, Schluter J, Foster KR (2015). The ecology of the microbiome: networks, competition, and stability. Sci.

[42] Poudel R, Jumpponen A, Schlatter DC, Paulitz TC, McSpadden Gardener BB, Kinkel LL (2016). Microbiome networks: a systems framework for identifying candidate microbial assemblages for disease management. Phytopathol.

[43] Bakker MG, Schlatter DC, Otto-Hanson L, Kinkel LL (2014). Diffuse symbioses: roles of plant–plant, plant–microbe and microbe–microbe interactions in structuring the soil microbiome. Mol Ecol.

[44] van der Heijden MG, Hartmann M (2016). Networking in the plant microbiome. PLOS Biol.

[45] Lau MK, Borrett SR, Baiser B, Gotelli NJ, Ellison AM (2017). Ecological network metrics: opportunities for synthesis. Ecosphere.

[46] Billick I, Case TJ (1994). Higher order interactions in ecological communities: what are they and how can they be detected?. Ecol.

[47] Carr A, Diener C, Baliga NS, Gibbons SM (2019). Use and abuse of correlation analyses in microbial ecology. ISME J.

[48] Vaz Jauri P, Bakker MG, Salomon CE, Kinkel LL (2013). Subinhibitory antibiotic concentrations mediate nutrient use and competition among soil *Streptomyces*. PLOS ONE.

[49] Borer ET, Harpole WS, Adler PB, Lind EM, Orrock JL, Seabloom EW (2014). Finding generality in ecology: a model for globally distributed experiments. Methods Ecol Evol.

[50] Borer ET, Grace JB, Harpole WS, MacDougall AS, Seabloom EW (2017). A decade of insights into grassland ecosystem responses to global environmental change. Nat Ecol Evol.

[51] Essarioui A, LeBlanc N, Kistler HC, Kinkel LL (2014). Plant host and community diversity impact the dynamics of resource use by soil *Streptomyces*. Phytopathol.

[52] LeBlanc N, Essarioui A, Kinkel LL, Kistler HC (2014). Fusarium community structure and carbon metabolism phenotypes respond to grassland plant community richness and plant host. Phytopathol.

[53] Essarioui A, Kistler HC, Kinkel LL (2016). Nutrient use preferences among soil *Streptomyces* suggest greater resource competition in monoculture than polyculture plant communities. Plant Soil.

[54] Essarioui A, LeBlanc N, Otto-Hanson L, Schlatter DC, Kistler HC, Kinkel LL (2020). Inhibitory and nutrient use phenotypes among coexisting fusarium and *Streptomyces* populations suggest local coevolutionary interactions in soil. Environ Microbiol.

[55] Schlatter D, Fubuh A, Xiao K, Hernandez D, Hobbie S, Kinkel L (2009). Resource amendments influence density and competitive phenotypes of *Streptomyces* in soil. Micro Ecol.

[56] Kinkel LL, Schlatter DC, Xiao K, Baines AD (2013). Sympatric inhibition and niche differentiation suggest alternative coevolutionary trajectories among *Streptomycetes*. ISME J.

[57] Reichardt J, Bornholdt S (2006). Statistical mechanics of community detection. Phys Rev E.

[58] Watts DJ, Strogatz SH (1998). Collective dynamics of ‘small-world’ networks. Nat.

[59] Allesina S, Levine JM (2011). A competitive network theory of species diversity. Proc Natl Acad Sci.

[60] Maynard DS, Bradford MA, Lindner DL, van Diepen LT, Frey SD, Glaeser JA (2017). Diversity begets diversity in competition for space. Nat Ecol Evol.

[61] Maynard DS, Crowther TW, Bradford MA (2017). Competitive network determines the direction of the diversity–function relationship. Proc Natl Acad Sci.

[62] Gallien L, Zimmermann NE, Levine JM, Adler PB (2017). The effects of intransitive competition on coexistence. Ecol Lett.

[63] Schlatter DC, Song Z, Vaz-Jauri P, Kinkel LL (2019). Inhibitory interaction networks among coevolved Streptomyces populations from prairie soils. PLOS ONE.

[64] Milo R (2002). Network motifs: simple building blocks of complex networks. Sci.

[65] Case TJ, Bender EA (1981). Testing for higher order interactions. Am Nat.

[66] Levine JM, Bascompte J, Adler PB, Allesina S (2017). Beyond pairwise mechanisms of species coexistence in complex communities. Nat.

[67] Mayfield MM, Stouffer DB (2017). Higher-order interactions capture unexplained complexity in diverse communities. Nat Ecol Evol.

[68] Friedman J, Higgins LM, Gore J (2017). Community structure follows simple assembly rules in microbial microcosms. Nat Ecol Evol.

[69] Bender EA, Canfield E (1978). The asymptotic number of labeled graphs with given degree sequences. J Comb Theory Ser A.

[70] Newman ME (2006). Modularity and community structure in networks. Proc Natl Acad Sci.

[71] Guo X, Boedicker JQ (2016). The contribution of high-order metabolic interactions to the global activity of a four-species microbial community. PLOS Comput Biol.

[72] Borrelli JJ, Allesina S, Amarasekare P, Arditi R, Chase I, Damuth J (2015). Selection on stability across ecological scales. Trends Ecol Evol.

[73] Davis GH, Crofoot MC, Farine DR (2018). Estimating the robustness and uncertainty of animal social networks using different observational methods. Anim Behav.

[74] Gilbertson ML, White LA, Craft ME (2020). Trade-offs with telemetry-derived contact networks for infectious disease studies in wildlife. Methods Ecol Evol.

[75] Grilli J, Barabás G, Michalska-Smith MJ, Allesina S (2017). Higher-order interactions stabilize dynamics in competitive network models. Nat.

[76] Letten AD, Stouffer DB (2019). The mechanistic basis for higher-order interactions and non-additivity in competitive communities. Ecol Lett.

[77] Dormann CF, Roxburgh SH (2005). Experimental evidence rejects pairwise modelling approach to coexistence in plant communities. Proc R Soc B Biol Sci.

[78] Staniczenko PP, Kopp JC, Allesina S (2013). The ghost of nestedness in ecological networks. Nat Commun.

[79] Großkopf T, Soyer OS (2014). Synthetic microbial communities. Curr Opin Microbiol.

[80] Holm S (1979). A simple sequentially rejective multiple test procedure. Scand J Stat.

