# Correction to: Network structure of resource use and niche overlap within the endophytic microbiome

**DOI:** 10.1038/s41396-021-01160-0

**Published:** 2021-11-30

**Authors:** Matthew Michalska-Smith, Zewei Song, Seth A. Spawn-Lee, Zoe A. Hansen, Mitch Johnson, Georgiana May, Elizabeth T. Borer, Eric W. Seabloom, Linda L. Kinkel

**Affiliations:** 1 Department of Veterinary Population Medicine, University of Minnesota, St Paul, MN USA; 2 Department of Plant Pathology, University of Minnesota, St Paul, MN USA; 3 Department of Geography, University of Wisconsin, Madison, WI USA; 4 Center for Sustainability and the Global Environment (SAGE), University of Wisconsin, Madison, WI USA; 5 Department of Microbiology & Molecular Genetics, Michigan State University, East Lansing, MI USA; 6 Department of Horticultural Science, University of Minnesota, St Paul, MN USA; 7 Department of Ecology, Evolution and Behavior, University of Minnesota, St Paul, MN USA

**Keywords:** Microbial ecology, Microbiome, Community ecology, Microbial ecology

Correction to: *The ISME Journal* 10.1038/s41396-021-01080-z, published online 19 August 2021

In this article were the solid coloured boxes that should be present in the table legends for 1, 3 and 4 have printed as hollow boxes. For table 5, there should be symbols in the legend as per the table but these have been replaced with hollow boxes.

The original article has been corrected.

