# Supplementary material for: Network structure of resource use and niche overlap within the endophytic microbiome
Source: ISME J. 2021 Aug 19;16(2):435–46. doi: 10.1038/s41396-021-01080-z (PMC8776778; doi:10.1038/s41396-021-01080-z)
Supplement: Supplementary file 1 — Supplementary Information [file 41396_2021_1080_MOESM1_ESM.pdf]

Supplementary Material

Taxonomy

Table S1: Number of unique families, genera, and isolates per order. There are 235 isolates in total.

kingdom	phylum	class	order	Number of unique families	genera	isolates
Bacteria	Actinobacteria	Actinobacteria	Actinomycetales	3	6	14
	Firmicutes	Bacilli	Bacillales	3	5	89
	Proteobacteria	Alphaproteobacteria	Rhizobiales	1	1	1
		Betaproteobacteria	Burkholderiales	1	1	1
		Gammaproteobacteria	Enterobacterales	2	3	9
			Pseudomonadales	1	1	1
Fungi	Ascomycota	Dothideomycetes	Capnodiales	1	1	1
			Pleosporales	1	1	3
		Eurotiomycetes	Eurotiales	1	2	58
		Sordariomycetes	Coniochaetales	1	1	1
			Glomerellales	1	1	3
			Hypocreales	4	7	45
			Phomatosporales	1	1	1
			Sordariales	1	1	1
			Xylariales	1	2	6
	Basidiomycota	Agaricomycetes	Polyporales	1	1	1

Table S2: Total number of unique taxonomic levels per microbial community.

treatment	leaf	Number of unique						
		kingdoms	phyla	classes	orders	families	genera	isolates
Control	1	2	3	4	4	5	6	20
	2	2	4	6	6	8	10	20
	3	2	3	4	4	4	5	20
	4	2	3	4	4	6	6	20
	5	2	3	3	3	4	4	15
	6	2	2	2	3	6	9	20
NPK Supplemented	1	2	4	6	7	10	10	20
	2	2	4	5	5	5	6	20
	3	2	3	5	6	6	8	20
	4	2	3	4	6	7	8	20
	5	2	2	4	4	5	5	20
	6	2	4	5	5	7	8	20
**Total:**		**2**	**5**	**9**	**16**	**24**	**35**	**235**

Alternative measures of niche overlap

Hypervolume overlap

In this section, we explore an alternative measure of niche overlap, in which we treat each isolate's growth on the 95 nutrients as a (maximally) 95-dimensional resource niche,

$$\eta(\vec{g}) = \left(\prod_m g_m\right)^{1/M},$$

where $\vec{g}$ is a vector of optical densities,m signifies a resource upon which isolate i had greater than 0 growth, and M signifies the total number of such resources. We then measure interactions by looking at the overlap of the partner isolate's hypervolume by one bounded in each direction by the lesser optical density between isolate i and the partner isolate j,

$$\Omega^{hv}_{i\to j} = \frac{\eta\left(\min_{1\leq n\leq N}\left(g_{i,n}, g_{j,n}\right)\right)}{\eta(\vec{g_j})}.$$

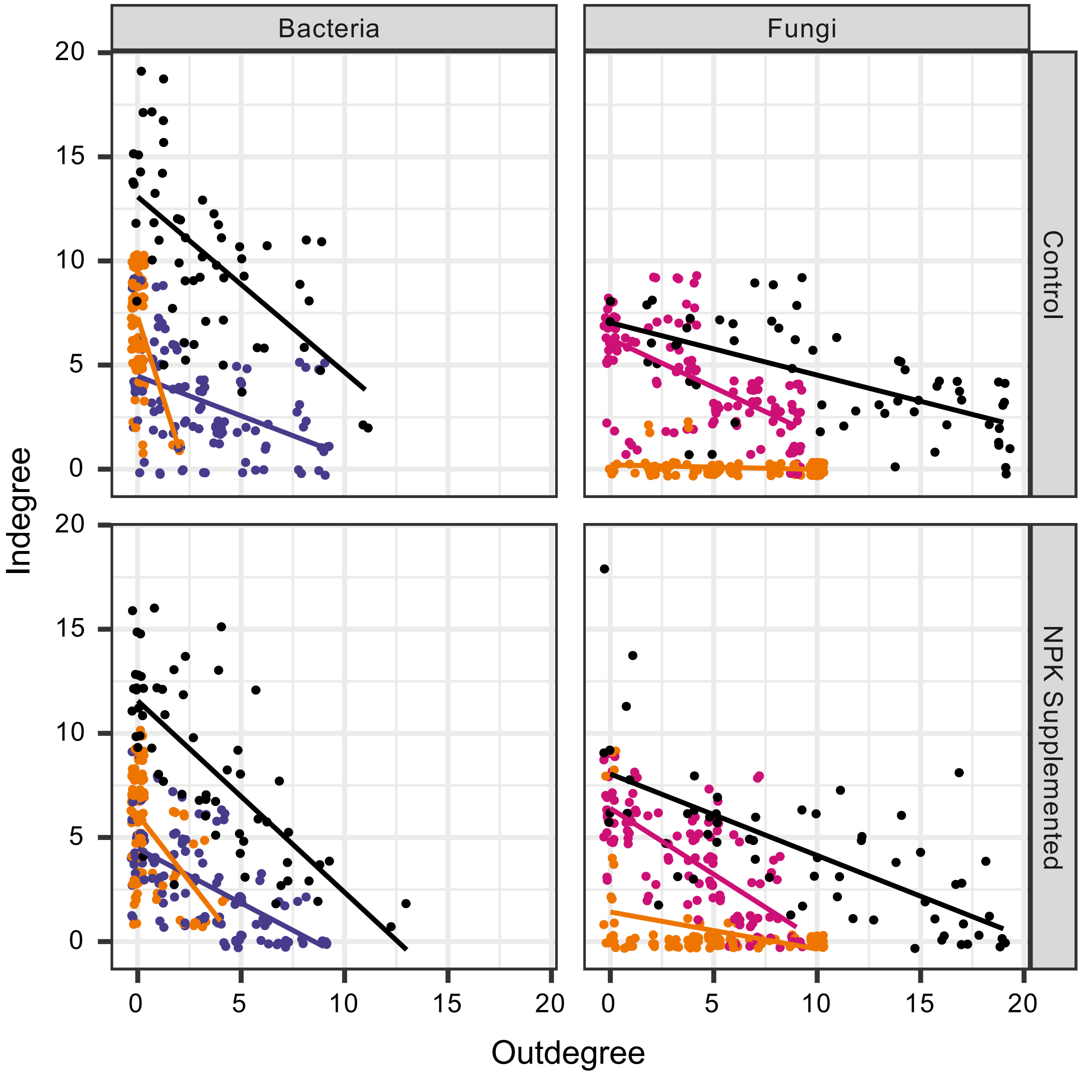

Figure S1: As Figure 3, but utilizing a hypervolume measure of niche overlap. Links are assigned when an isolate has greater than 82 % niche overlap with another isolate. Points do not fall exactly on integers due to slight jittering to improve readability. Colored points indicate the kingdom of the partner isolate: orange indicates interactions between kingdoms, while within kingdom links are colored according to the kingdom, using the same colors as in Figure 2 (pink for fungi, blue for bacteria). Black points indicate values when partner's kingdom is ignored. Thus, there are three points for each isolate in each panel: one for their interactions with other members of their same kingdom (blue or pink for bacteria or fungi, respectively), one for interactions with the alternate kingdom (orange), and one for their total number of interactions (black). Lines indicate best fitting linear models for each subset of the data. Slopes are significantly different from 0 in all cases except for the Bacteria → Bacteria interaction in the control treatment (blue points in top-left panel; Table S3).

Table S3: Linear model results for indegree by outdegree in each sympatric network, differentiated according to focal and partner isolates' Kingdom (each unique combination of color and panel in Figure S1). This analysis utilizes a hypervolume measure of niche overlap and binary interaction strengths. An analysis of variance in these slopes is presented in Table S4.

Treatment	Focal Kingdom	Partner Kingdom	Estimate	p value	Adjusted R^2
Control	Bacteria	Any	−0.84	< 0.001	0.37
		Bacteria	−0.38	< 0.001	0.18
		Fungi	−3.14	< 0.001	0.20
	Fungi	Any	−0.25	< 0.001	0.37
		Bacteria	−0.02	0.030	0.03
		Fungi	−0.47	< 0.001	0.40
NPK Supplemented	Bacteria	Any	−0.92	< 0.001	0.53
		Bacteria	−0.52	< 0.001	0.39
		Fungi	−1.27	< 0.001	0.26
	Fungi	Any	−0.39	< 0.001	0.47
		Bacteria	−0.18	< 0.001	0.15
		Fungi	−0.63	< 0.001	0.49

Table S4: Summary of an analysis of variance for linear models relating isolate outdegree to indegree using a hypervolume measure of niche overlap and binary interaction strengths (reported in Table S3).

Term	df	Sum squared error	Mean squared error	p value
Outdegree	1	3528.14	3528.14	< 0.001
Treatment	1	125.40	125.40	< 0.001
Focal Kingdom	1	695.81	695.81	< 0.001
Partner Kingdom	2	5678.59	2839.29	< 0.001
Treatment:Focal Kingdom	1	44.09	44.09	0.003
Treatment:Partner Kingdom	2	45.28	22.64	0.010
Residuals	1166	5741.53	4.92	NA
R^2	0.64			

Vector projection

In this section, we explore an alternative measure of niche overlap, in which we treat each isolate's growth on the 95 nutrients as a vector in 95-dimensional space, whose magnitude can be calculated as

$$|\vec{g}| = \sqrt{\vec{g} \times \vec{g}^T}.$$

We then measure interactions by looking at how much an isolate's resource use vector is overlapped by a projection of another isolate's resource use vector into the same direction,

$$\Omega_{i \to j}^{vp} = \frac{\vec{g_i} \times \frac{\vec{g_j}}{|\vec{g_j}|}}{|\vec{g_j}|}.$$

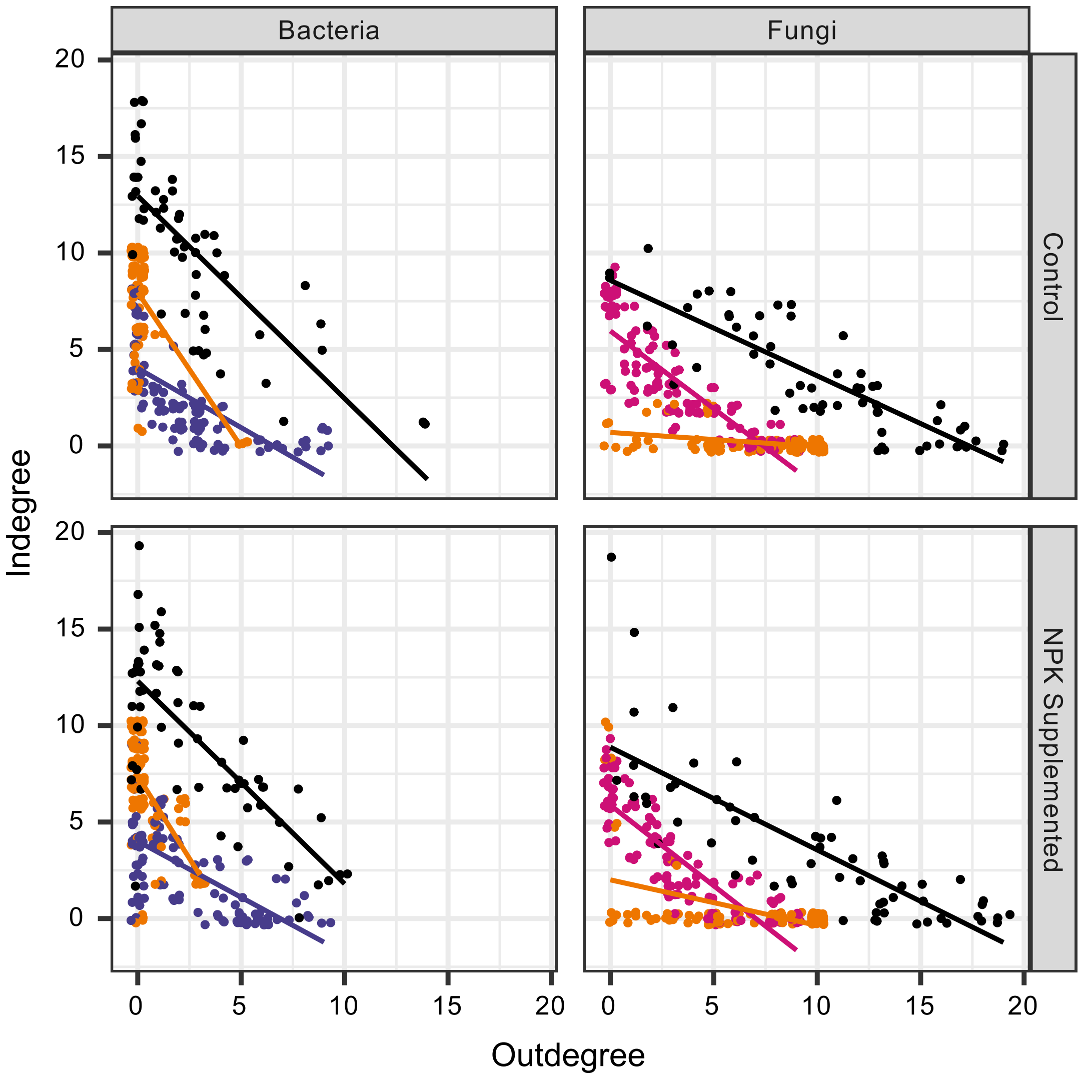

Figure S2: As Figure 3, but utilizing a vector projection measure of niche overlap. Links are assigned when an isolate has 100 % niche overlap with another isolate. Points do not fall exactly on integers due to slight jittering to improve readability. Colored points indicate the kingdom of the partner isolate: orange indicates interactions between kingdoms, while within kingdom links are colored according to the kingdom, using the same colors as in Figure 2 (pink for fungi, blue for bacteria). Black points indicate values when partner's kingdom is ignored. Thus, there are three points for each isolate in each panel: one for their interactions with other members of their same kingdom (blue or pink for bacteria or fungi, respectively), one for interactions with the alternate kingdom (orange), and one for their total number of interactions (black). Lines indicate best fitting linear models for each subset of the data. All slopes are significantly different from 0 (Table S5).

Table S5: Linear model results for indegree by outdegree in each sympatric network, differentiated according to focal and partner isolates' Kingdom (each unique combination of color and panel in Figure S2). This analysis utilizes a vector projection measure of niche overlap and binary interaction strengths. An analysis of variance in these slopes is presented in Table S6.

Treatment	Focal Kingdom	Partner Kingdom	Estimate	p value	Adjusted R^2
Control	Bacteria	Any	-1.05	< 0.001	0.59
		Bacteria	-0.62	< 0.001	0.44
		Fungi	-1.60	< 0.001	0.28
	Fungi	Any	-0.49	< 0.001	0.71
		Bacteria	-0.07	< 0.001	0.14
		Fungi	-0.81	< 0.001	0.71
NPK Supplemented	Bacteria	Any	-1.05	< 0.001	0.57
		Bacteria	-0.58	< 0.001	0.44
		Fungi	-1.71	< 0.001	0.36
	Fungi	Any	-0.53	< 0.001	0.63
		Bacteria	-0.24	< 0.001	0.20
		Fungi	-0.84	< 0.001	0.69

Table S6: Summary of an analysis of variance for linear models relating isolate outdegree to indegree using a vector projection measure of niche overlap and binary interaction strengths (reported in Table S5).

Term	df	Sum squared error	Mean squared error	p value
Outdegree	1	6566.86	6566.86	< 0.001
Treatment	1	16.49	16.49	0.060
Focal Kingdom	1	578.36	578.36	< 0.001
Partner Kingdom	2	5811.98	2905.99	< 0.001
Treatment:Focal Kingdom	1	12.01	12.01	0.108
Treatment:Partner Kingdom	2	16.57	8.28	0.168
Residuals	1166	5405.05	4.64	NA
R^2	0.71			

Weighted interactions

Average pairwise overlap

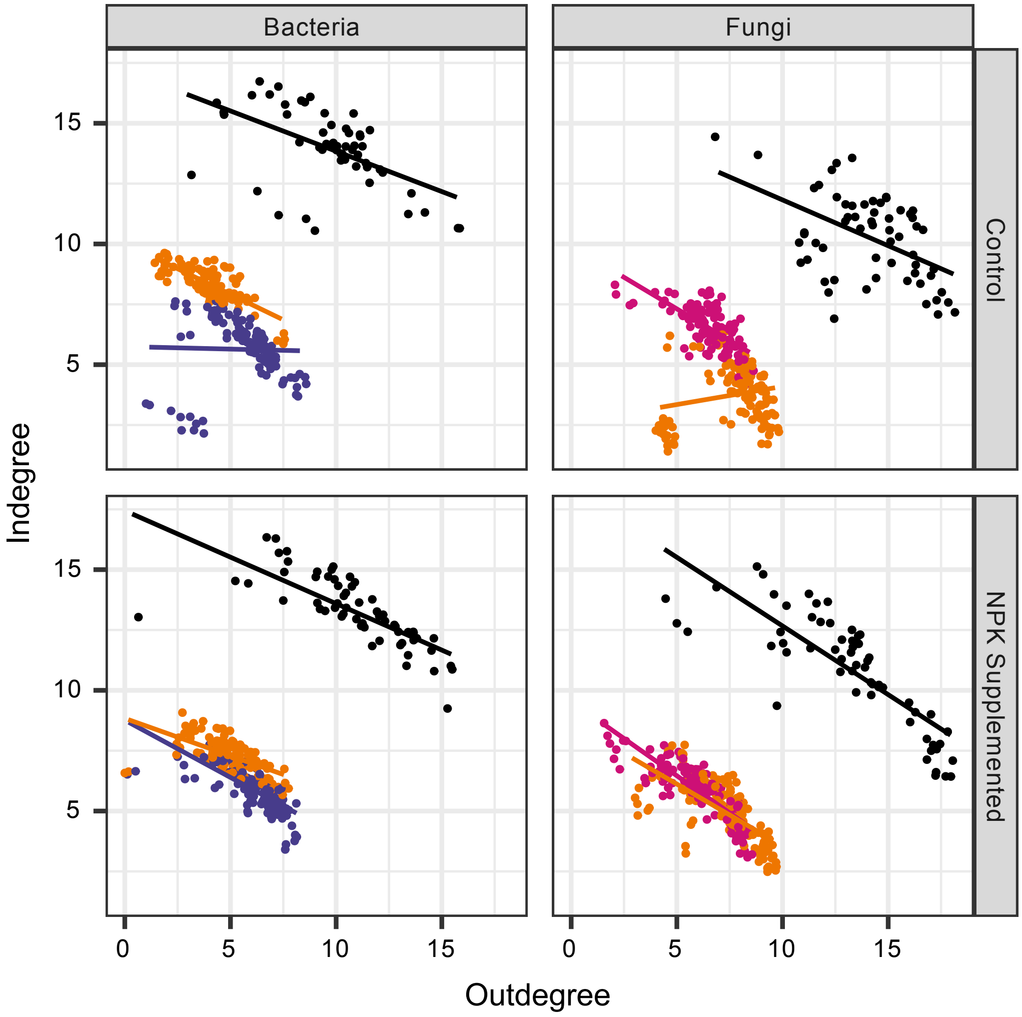

Figure S3: As Figure 3, but incorporating weighted interactions. Colored points indicate the kingdom of the partner isolate: orange indicates interactions between kingdoms, while within kingdom links are colored according to the kingdom, using the same colors as in Figure 2 (pink for fungi, blue for bacteria). Black points indicate values when partner's kingdom is ignored. Thus, there are three points for each isolate in each panel: one for their interactions with other members of their same kingdom (blue or pink for bacteria or fungi, respectively), one for interactions with the alternate kingdom (orange), and one for their total number of interactions (black). Lines indicate best fitting linear models for each subset of the data. Slopes are significantly different from 0 in all cases except for the Bacteria → Bacteria interaction in the control treatment (blue points in top-left panel; Table S7). Removal of the outlying control network (see main text) results in all slopes being significant and a sign reversal in the case of Fungi → Bacteria interactions (orange points in top-right panel)

Table S7: Linear model results for indegree by outdegree in each sympatric network, differentiated according to focal and partner isolates' Kingdom (each unique combination of color and panel in Figure S3). This analysis utilizes an average pairwise measure of niche overlap and weighted interaction strengths. An analysis of variance in these slopes is presented in Table S8.

Treatment	Focal Kingdom	Partner Kingdom	Estimate	p value	Adjusted R^2
Control	Bacteria	Any	−0.33	< 0.001	0.29
		Bacteria	−0.02	0.791	−0.01
		Fungi	−0.42	< 0.001	0.65
	Fungi	Any	−0.38	< 0.001	0.24
		Bacteria	0.15	0.025	0.03
		Fungi	−0.52	< 0.001	0.49
NPK Supplemented	Bacteria	Any	−0.38	< 0.001	0.55
		Bacteria	−0.47	< 0.001	0.59
		Fungi	−0.31	< 0.001	0.37
	Fungi	Any	−0.57	< 0.001	0.67
		Bacteria	−0.51	< 0.001	0.48
		Fungi	−0.62	< 0.001	0.77

Table S8: Summary of an analysis of variance for linear models relating isolate outdegree to indegree using an average pairwise measure of niche overlap and weighted interaction strengths (reported in Table S7).

Term	df	Sum squared error	Mean squared error	p value
Outdegree	1	983.02	983.02	< 0.001
Treatment	1	0.05	0.05	0.830
Focal Kingdom	1	1843.86	1843.86	< 0.001
Partner Kingdom	2	6490.50	3245.25	< 0.001
Treatment:Focal Kingdom	1	5.00	5.00	0.029
Treatment:Partner Kingdom	2	135.44	67.72	< 0.001
Residuals	1166	1220.86	1.05	NA
R^2	0.89			

Vector projection

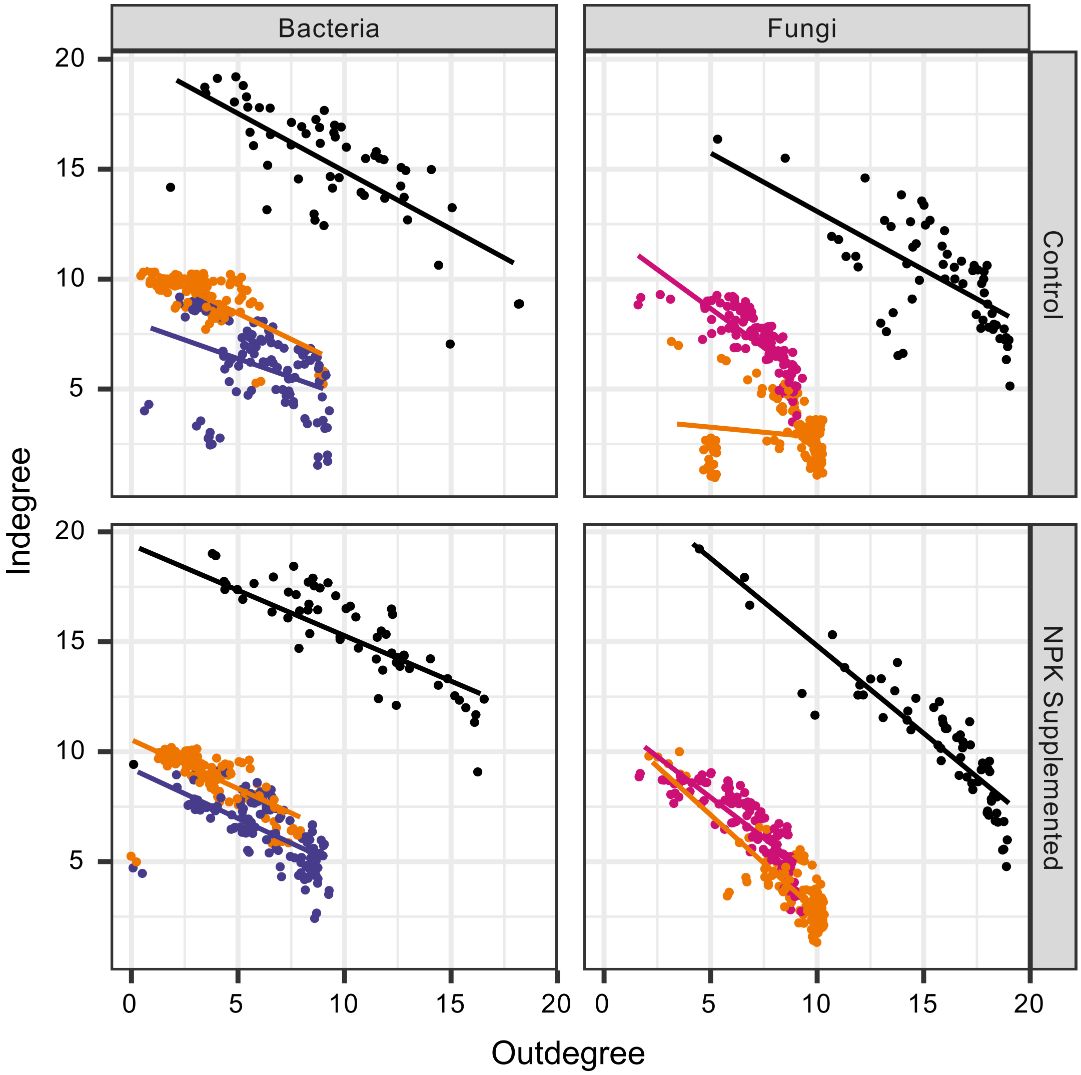

Figure S4: As Figure 3, but utilizing a vector projection measure of niche overlap and weighted interactions. Colored points indicate the kingdom of the focal isolate: orange indicates interactions between kingdoms, while within kingdom links are colored according to the kingdom, using the same colors as in Figure 2 (pink for fungi, blue for bacteria). Black points indicate values when the partner's kingdom is ignored. Thus, there are three points for each isolate in each panel: one for their interactions with other members of their same kingdom (blue or pink for bacteria or fungi, respectively), one for interactions with the alternate kingdom (orange), and one for their total number of interactions (black). Lines indicate best fitting linear models for each subset of the data. All slopes are significantly different from 0 except for Fungi → Bacteria interactions in the control treatment (orange points in top-right panel; Table S9). Removal of the outlying control network (see main text) results in all slopes being significant.

Table S9: Linear model results for indegree by outdegree in each sympatric network, differentiated according to focal and partner isolates' Kingdom (each unique combination of color and panel in Figure S4). This analysis utilizes a vector projection measure of niche overlap and weighted interaction strengths. An analysis of variance in these slopes is presented in Table S10.

Treatment	Focal Kingdom	Partner Kingdom	Estimate	p value	Adjusted R^2
Control	Bacteria	Any	−0.53	< 0.001	0.52
		Bacteria	−0.34	< 0.001	0.13
		Fungi	−0.47	< 0.001	0.55
	Fungi	Any	−0.53	< 0.001	0.41
		Bacteria	−0.09	0.134	0.01
		Fungi	−0.71	< 0.001	0.66
NPK Supplemented	Bacteria	Any	−0.41	< 0.001	0.44
		Bacteria	−0.45	< 0.001	0.38
		Fungi	−0.44	< 0.001	0.45
	Fungi	Any	−0.80	< 0.001	0.83
		Bacteria	−0.88	< 0.001	0.75
		Fungi	−0.74	< 0.001	0.74

Table S10: Summary of an analysis of variance for linear models relating isolate outdegree to indegree using a vector projection measure of niche overlap and weighted interaction strengths (reported in Table S9).

Term	df	Sum squared error	Mean squared error	p value
Outdegree	1	19.51	19.51	< 0.001
Treatment	1	6.38	6.38	0.058
Focal Kingdom	1	3098.68	3098.68	< 0.001
Partner Kingdom	2	11357.62	5678.81	< 0.001
Treatment:Focal Kingdom	1	0.34	0.34	0.662
Treatment:Partner Kingdom	2	84.46	42.23	< 0.001
Residuals	1166	2069.23	1.77	NA
R^2	0.88			

Hypervolume overlap

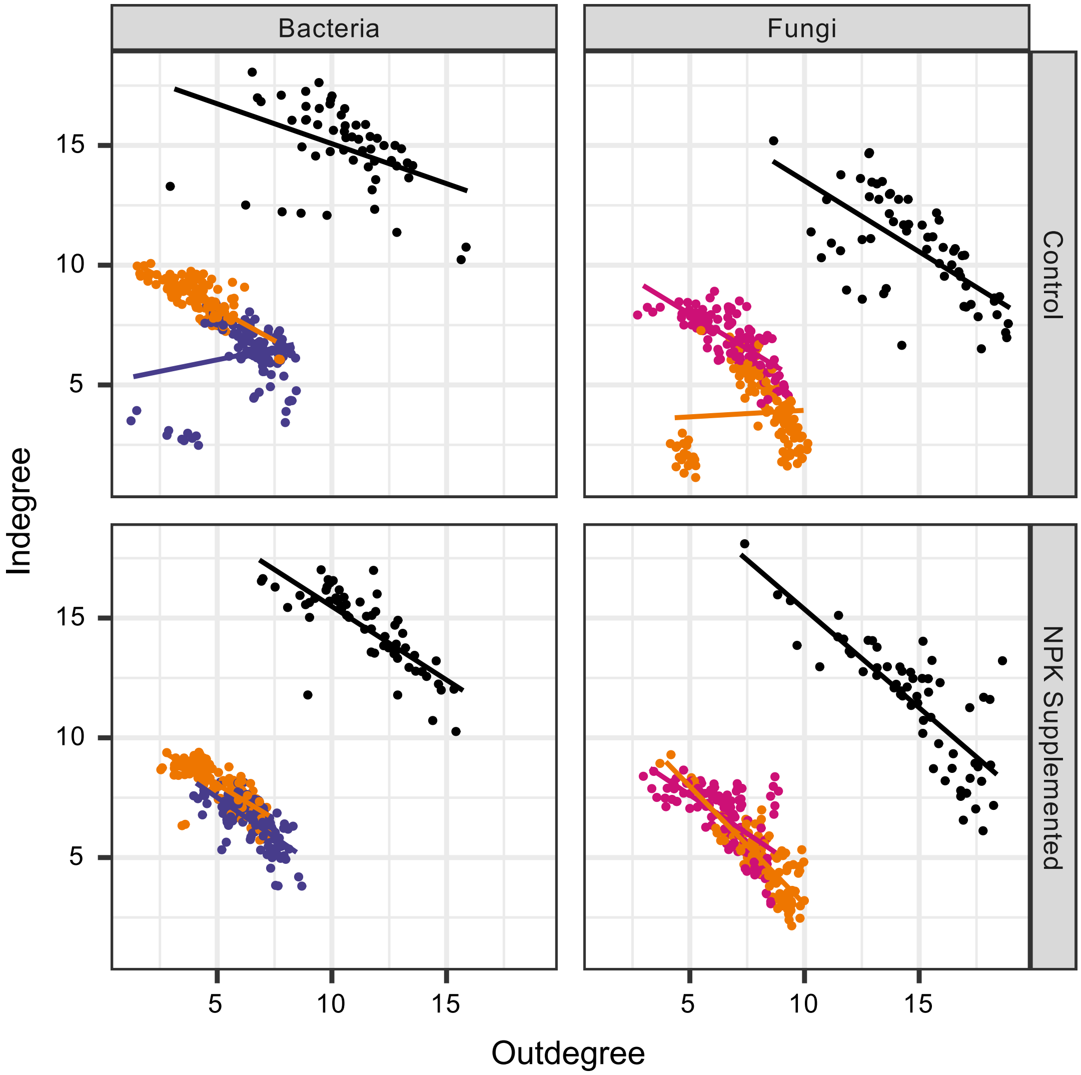

Figure S5: As Figure 3, but utilizing a hypervolume measure of niche overlap and weighted interactions. Colored points indicate the kingdom of the partner isolate: orange indicates interactions between kingdoms, while within kingdom links are colored according to the kingdom, using the same colors as in Figure 2 (pink for fungi, blue for bacteria). Black points indicate values when the partner's kingdom is ignored. Thus, there are three points for each isolate in each panel: one for their interactions with other members of their same kingdom (blue or pink for bacteria or fungi, respectively), one for interactions with the alternate kingdom (orange), and one for their total number of interactions (black). Lines indicate best fitting linear models for each subset of the data. Slopes are significantly different from 0 in all cases except for the Fungi → Bacteria interaction in the control treatment (orange points in top-right panel; Table S11). Removal of the outlying control network (see main text) results in all slopes being significant and a sign reversal in the case of Bacteria → Bacteria interactions (blue points in top-left panel).

Table S11: Linear model results for indegree by outdegree in each sympatric network, differentiated according to focal and partner isolates' Kingdom (each unique combination of color and panel in Figure S5). This analysis utilizes a hypervolume measure of niche overlap and weighted interaction strengths. An analysis of variance in these slopes is presented in Table S12.

Treatment	Focal Kingdom	Partner Kingdom	Estimate	p value	Adjusted R^2
Control	Bacteria	Any	−0.33	< 0.001	0.18
		Bacteria	0.19	0.038	0.03
		Fungi	−0.53	< 0.001	0.64
	Fungi	Any	−0.59	< 0.001	0.43
		Bacteria	0.05	0.504	−0.00
		Fungi	−0.58	< 0.001	0.58
NPK Supplemented	Bacteria	Any	−0.61	< 0.001	0.63
		Bacteria	−0.66	< 0.001	0.49
		Fungi	−0.58	< 0.001	0.64
	Fungi	Any	−0.82	< 0.001	0.64
		Bacteria	−0.98	< 0.001	0.72
		Fungi	−0.64	< 0.001	0.43

Table S12: Summary of an analysis of variance for linear models relating isolate outdegree to indegree using a hypervolume measure of niche overlap and weighted interaction strengths (reported in Table S11).

Term	df	Sum squared error	Mean squared error	p value
Outdegree	1	1224.68	1224.68	< 0.001
Treatment	1	6.03	6.03	0.042
Focal Kingdom	1	2750.62	2750.62	< 0.001
Partner Kingdom	2	7141.08	3570.54	< 0.001
Treatment:Focal Kingdom	1	9.40	9.40	0.011
Treatment:Partner Kingdom	2	69.34	34.67	< 0.001
Residuals	1166	1694.63	1.45	NA
R^2	0.87			

Alternative cutoff for binary link definition

Cutoff of 0.5

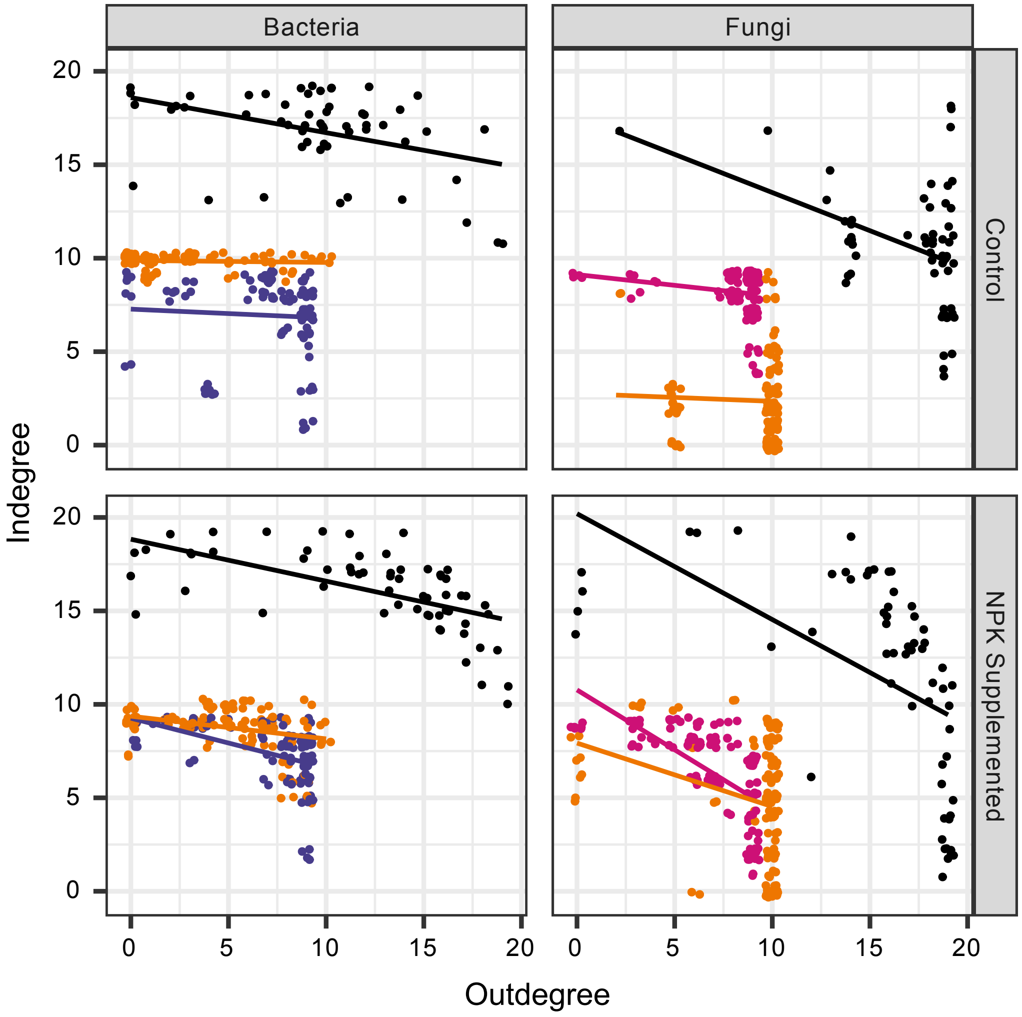

Figure S6: As Figure 3, but incorporating using an alternative cutoff of 0.5 to define link presence/absence. Colored points indicate the kingdom of the partner isolate: orange indicates interactions between kingdoms, while within kingdom links are colored according to the kingdom, using the same colors as in Figure 2 (pink for fungi, blue for bacteria). Black points indicate values when partner's kingdom is ignored. Thus, there are three points for each isolate in each panel: one for their interactions with other members of their same kingdom (blue or pink for bacteria or fungi, respectively), one for interactions with the alternate kingdom (orange), and one for their total number of interactions (black). Lines indicate best fitting linear models for each subset of the data. Slopes are significantly different from 0 except for three cases in the control treatment: Fungi → Bacteria interactions (orange points in the top-right panel), Bacteria → Bacteria interaction (blue points in top-left panel), and Bacteria → Fungi interactions (orange points in the top-left panel; Table S7). Removal of the outlying control network (see main text) results in the two former cases becoming significant as well.

Table S13: Linear model results for indegree by outdegree in each sympatric network, differentiated according to focal and partner isolates' Kingdom (each unique combination of color and panel in Figure S6). This analysis utilizes an average pairwise measure of niche overlap and binary interaction strengths. An analysis of variance in these slopes is presented in Table S14.

Treatment	Focal Kingdom	Partner Kingdom	Estimate	p value	Adjusted R^2
Control	Bacteria	Any	−0.19	0.003	0.14
		Bacteria	−0.05	0.525	−0.01
		Fungi	−0.01	0.342	−0.00
	Fungi	Any	−0.41	0.003	0.13
		Bacteria	−0.04	0.698	−0.01
		Fungi	−0.11	0.046	0.03
NPK Supplemented	Bacteria	Any	−0.22	< 0.001	0.34
		Bacteria	−0.27	< 0.001	0.21
		Fungi	−0.12	< 0.001	0.10
	Fungi	Any	−0.57	< 0.001	0.28
		Bacteria	−0.34	< 0.001	0.08
		Fungi	−0.64	< 0.001	0.44

Table S14: Summary of an analysis of variance for linear models relating isolate outdegree to indegree using an average pairwise measure of niche overlap and binary interaction strengths (reported in Table S13).

Term	df	Sum squared error	Mean squared error	p value
Outdegree	1	22.71	22.71	0.042
Treatment	1	6.33	6.33	0.283
Focal Kingdom	1	3660.70	3660.70	< 0.001
Partner Kingdom	2	11077.53	5538.77	< 0.001
Treatment:Focal Kingdom	1	6.01	6.01	0.296
Treatment:Partner Kingdom	2	469.30	234.65	< 0.001
Residuals	1166	6405.57	5.49	NA
R^2	0.70			

Table S15: As Table 5, but incorporating using an alternative cutoff of 0.5 to define link presence/absence. Summary test for higher-order interactions in endophytic microbial networks. Empirical p values (left) and z scores (right) for network structure metric comparisons between empirical and randomly-rewired networks. Each column represents an individual leaf, ordered as in Figure 2. Values less than 0.05 (-2) signify that the empirical value is significantly smaller than expected, and are represented by ⌄ (⌄⌄ for values less than 0.01 (-4)). Likewise, values greater than 0.95 (2) signify that the empirical value is larger than expected and are represented by ⌃ (⌃⌃ for values greater than 0.99 (4)). Dashes (–) signify values between 0.05 and 0.95 (-2 and 2 ; *i.e.* non-significant differences) and × indicates cases where all randomizations resulted in the same value for this metric/community combination. Note that, for this cutoff, all networks had sufficient unique configurations to be included.

	p value										z score									
Metric/Triad	Control				NPK Supplemented						Control				NPK Supplemented					
Network Clustering	–	–	–	⌄⌄	⌃⌃	–	–	–	⌃⌃	–	–	–	–	⌄	⌃	–	–	–	⌃⌃	–
Intransitivity	–	×	–	⌃⌃	–	–	–	×	–	×	–	×	–	⌃	–	–	–	×	–	×
1.	–	–	–	–	⌄⌄	–	⌄	–	⌄⌄	–	–	–	–	–	⌄	–	–	–	⌄	–
2.	–	–	–	–	⌃⌃	–	⌃⌃	–	⌃⌃	–	–	–	–	–	⌃	–	⌃	–	⌃⌃	–
3.	–	×	–	⌃⌃	–	–	⌃	×	–	×	–	×	–	⌃⌃	–	–	⌃	×	–	×
4.	–	–	–	–	⌄⌄	⌄	–	–	⌄⌄	–	–	–	–	–	⌄	–	–	–	⌄⌄	–
5.	–	–	–	⌃⌃	⌄⌄	–	–	–	⌄⌄	–	–	–	–	⌃	⌄	–	–	–	⌄⌄	–
6.	–	–	–	–	⌄	–	–	–	⌄⌄	–	–	–	–	–	⌄	–	–	–	⌄	–
7.	–	×	–	–	–	–	–	×	–	×	–	×	–	–	–	–	–	×	–	×
8.	–	×	–	–	–	–	–	×	–	×	⌃	×	–	–	–	–	–	×	–	×
9.	–	–	–	⌄⌄	⌃⌃	–	–	–	⌃⌃	–	–	–	–	⌄	–	–	–	–	⌃	–
10.	×	×	×	–	–	–	–	×	–	×	×	×	×	–	–	–	–	×	–	×
11.	×	×	×	–	×	–	–	×	×	×	×	×	×	–	×	–	–	×	×	×
12.	–	×	–	–	–	–	–	×	–	×	–	×	–	–	–	–	–	×	–	×
13.	–	×	–	⌃⌃	–	–	⌃	×	–	×	–	×	–	⌃	–	–	–	×	–	×
14.	–	×	×	–	–	–	–	×	–	×	–	×	×	–	–	–	–	×	–	×
15.	–	×	×	–	×	–	⌃	×	×	×	–	×	×	–	×	–	⌃⌃	×	×	×
16.	×	×	×	⌃⌃	×	×	×	×	×	×	×	×	×	⌃⌃	×	×	×	×	×	×

Cutoff of 0.875

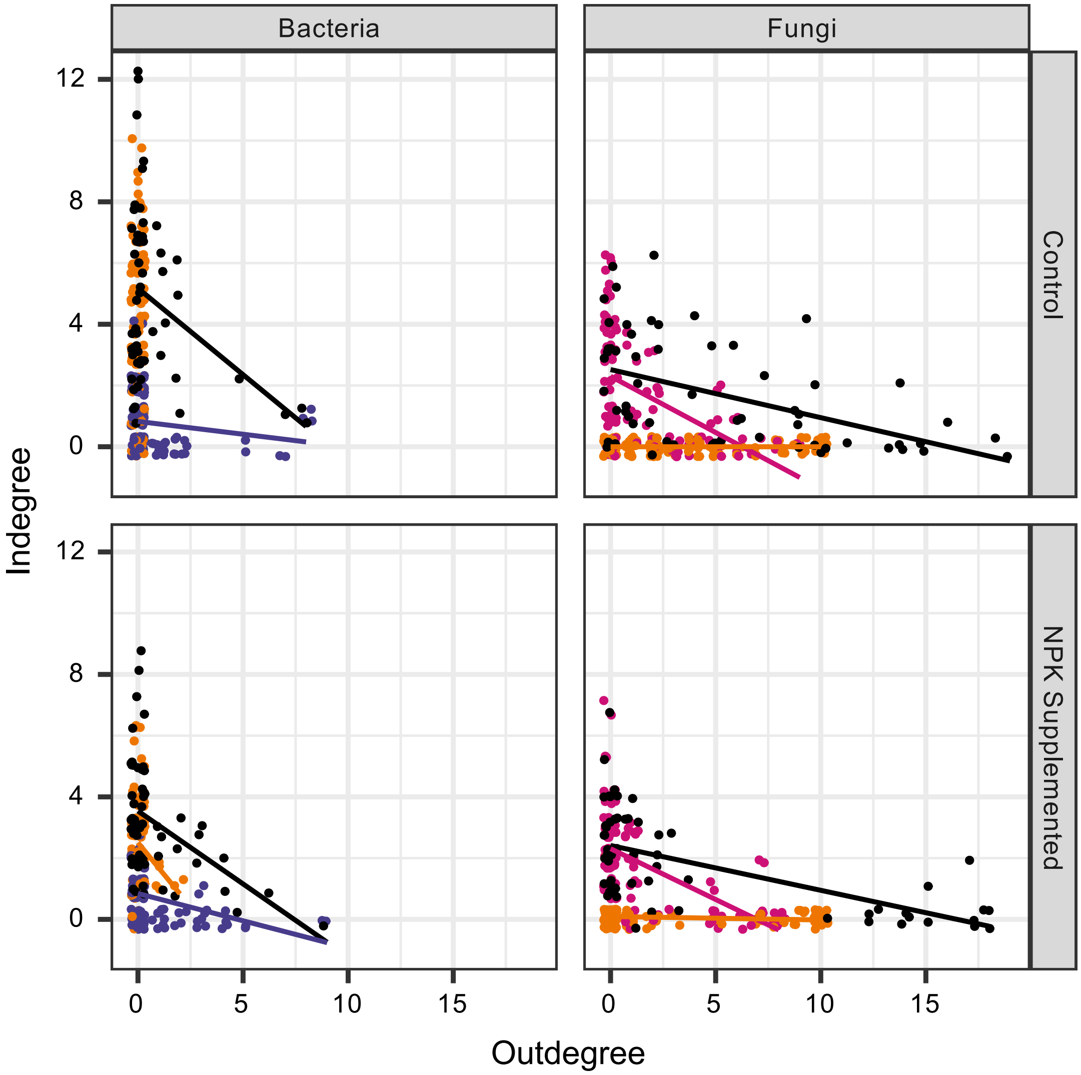

Figure S7: As Figure 3, but incorporating using an alternative cutoff of 0.875 to define link presence/absence. Colored points indicate the kingdom of the partner isolate: orange indicates interactions between kingdoms, while within kingdom links are colored according to the kingdom, using the same colors as in Figure 2 (pink for fungi, blue for bacteria). Black points indicate values when partner's kingdom is ignored. Thus, there are three points for each isolate in each panel: one for their interactions with other members of their same kingdom (blue or pink for bacteria or fungi, respectively), one for interactions with the alternate kingdom (orange), and one for their total number of interactions (black). Lines indicate best fitting linear models for each subset of the data. Slopes are significantly different from 0 in all cases except for the Bacteria → Bacteria interaction in the control treatment (blue points in top-left panel) and the Bacteria → Fungi interactions in the NPK-supplemented treatment (orange points in the bottom-left panel; Table S7). Note also that there were no Bacteria → Fungi interactions in the control treatment.

Table S16: Linear model results for indegree by outdegree in each sympatric network, differentiated according to focal and partner isolates' Kingdom (each unique combination of color and panel in Figure S7). This analysis utilizes an average pairwise measure of niche overlap and binary interaction strengths. An analysis of variance in these slopes is presented in Table S17.

Treatment	Focal Kingdom	Partner Kingdom	Estimate	p value	Adjusted R^2
Control	Bacteria	Any	−0.56	0.005	0.12
		Bacteria	−0.08	0.074	0.02
		Fungi	0.00	NA	NA
	Fungi	Any	−0.16	< 0.001	0.23
		Bacteria	0.00	NA	NA
		Fungi	−0.37	< 0.001	0.24
NPK Supplemented	Bacteria	Any	−0.48	< 0.001	0.19
		Bacteria	−0.18	< 0.001	0.10
		Fungi	−0.84	0.032	0.03
	Fungi	Any	−0.15	< 0.001	0.35
		Bacteria	−0.01	0.191	0.01
		Fungi	−0.33	< 0.001	0.35

Table S17: Summary of an analysis of variance for linear models relating isolate outdegree to indegree using an average pairwise measure of niche overlap and binary interaction strengths (reported in Table S16).

Term	df	Sum squared error	Mean squared error	p value
Outdegree	1	539.52	539.52	< 0.001
Treatment	1	68.22	68.22	< 0.001
Focal Kingdom	1	224.14	224.14	< 0.001
Partner Kingdom	2	1335.13	667.56	< 0.001
Treatment:Focal Kingdom	1	60.61	60.61	< 0.001
Treatment:Partner Kingdom	2	28.76	14.38	0.002
Residuals	1166	2605.12	2.23	NA
R^2	0.46			

Table S18: As Table 5, but incorporating using an alternative cutoff of 0.875 to define link presence/absence. Summary test for higher-order interactions in endophytic microbial networks. Empirical p values (left) and z scores (right) for network structure metric comparisons between empirical and randomly-rewired networks. Each column represents an individual leaf, ordered as in Figure 2. Values less than 0.05 (-2) signify that the empirical value is significantly smaller than expected, and are represented by ⌄ (⩔ for values less than 0.01 (-4)). Likewise, values greater than 0.95 (2) signify that the empirical value is larger than expected and are represented by ⌃ (⩓ for values greater than 0.99 (4)). Dashes (–) signify values between 0.05 and 0.95 (-2 and 2 ; *i.e.* non-significant differences) and × indicates cases where all randomizations resulted in the same value for this metric/community combination. Note that, for this cutoff, all of the control leaves and two of the NPK leaves (N1 and N6) were omitted from this analysis due to a lack of unique network configurations.

	p value		z score	
Metric/Triad	Control	NPK Supplemented	Control	NPK Supplemented
Network Clustering	– – – ⩔	⩓ – – – ⩓ –	– – – ⌄	⌃ – – – ⩓ –
Intransitivity	– × – ⩓	– – – × – ×	– × – ⌃	– – – × – ×
1. 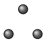	– – – –	⩔ – ⌄ – ⩔ –	– – – –	⌄ – – – ⌄ –
2. 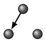	– – – –	⩓ – ⩓ – ⩓ –	– – – –	⌃ – ⌃ – ⩓ –
3. 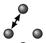	– × – ⩓	– – ⌃ × – ×	– × – ⩓	– – ⌃ × – ×
4. 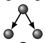	– – – –	⩔ ⌄ – – ⩔ –	– – – –	⌄ – – – ⩔ –
5. 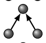	– – – ⩓	⩔ – – – ⩔ –	– – – ⌃	⌄ – – – ⩔ –
6. 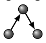	– – – –	⌄ – – – ⩔ –	– – – –	⌄ – – – ⌄ –
7. 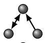	– × – –	– – – × – ×	– × – –	– – – × – ×
8. 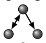	– × – –	– – – × – ×	⌃ × – –	– – – × – ×
9. 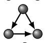	– – – ⩔	⩓ – – – ⩓ –	– – – ⌄	– – – – ⌃ –
10. 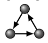	× × × –	– – – × – ×	× × × –	– – – × – ×
11. 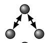	× × × –	× – – × × ×	× × × –	× – – × × ×
12. 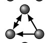	– × – –	– – – × – ×	– × – –	– – – × – ×
13. 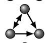	– × – ⩓	– – ⌃ × – ×	– × – ⌃	– – – × – ×
14. 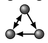	– × × –	– – – × – ×	– × × –	– – – × – ×
15. 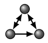	– × × –	× – ⌃ × × ×	– × × –	× – ⩓ × × ×
16. 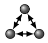	× × × ⩓	× × × × × ×	× × × ⩓	× × × × × ×

Additional tables relating to main text figures

Table S19: Statistical differences in mean (Welch's t-Test) and shape (Kolmogorov–Smirnov test) between treatments in degree histograms from Figure 1.

Interaction Type	NPK-supplemented	Control	T-test p value	KS-test p value
Bacteria → Bacteria	0.661	0.659	0.896	0.116
Bacteria → Fungi	0.494	0.408	< 0.001	< 0.001
Fungi → Bacteria	0.731	0.831	< 0.001	< 0.001
Fungi → Fungi	0.656	0.724	< 0.001	< 0.001

Table S20: χ^2-test results comparing the distribution of isolate kingdom across node groupings found using a spinglass algorithm (57). A filled box signifies a significant p value, *i.e.* a case where the distribution of kingdoms across groups is significantly non-uniform, with the intensity of the color indicating the α level of the significance ■ and ▦ corresponding to < 0.001 and < 0.01, respectively). The fifth binary control network did not divide into two groups with more than one isolate in each, and thus was ineligible for a χ^2 analysis.

Weights	Control	NPK Supplemented
Binary	□□□□×□	▦□■□■▦
Weighted	■■■■■■	■■▦■▦▦

Table S21: Summary of an analysis of variance for linear models relating isolate outdegree to indegree using an average pairwise measure of niche overlap and binary interaction strengths (reported in Table 2).

Term	df	Sum squared error	Mean squared error	p value
Outdegree	1	4376.80	4376.80	< 0.001
Treatment	1	266.62	266.62	< 0.001
Focal Kingdom	1	838.89	838.89	< 0.001
Partner Kingdom	2	5847.50	2923.75	< 0.001
Treatment:Focal Kingdom	1	1.28	1.28	0.571
Treatment:Partner Kingdom	2	310.22	155.11	< 0.001
Residuals	1166	4649.99	3.99	NA
R^2	0.71			

Taxomomic Analysis

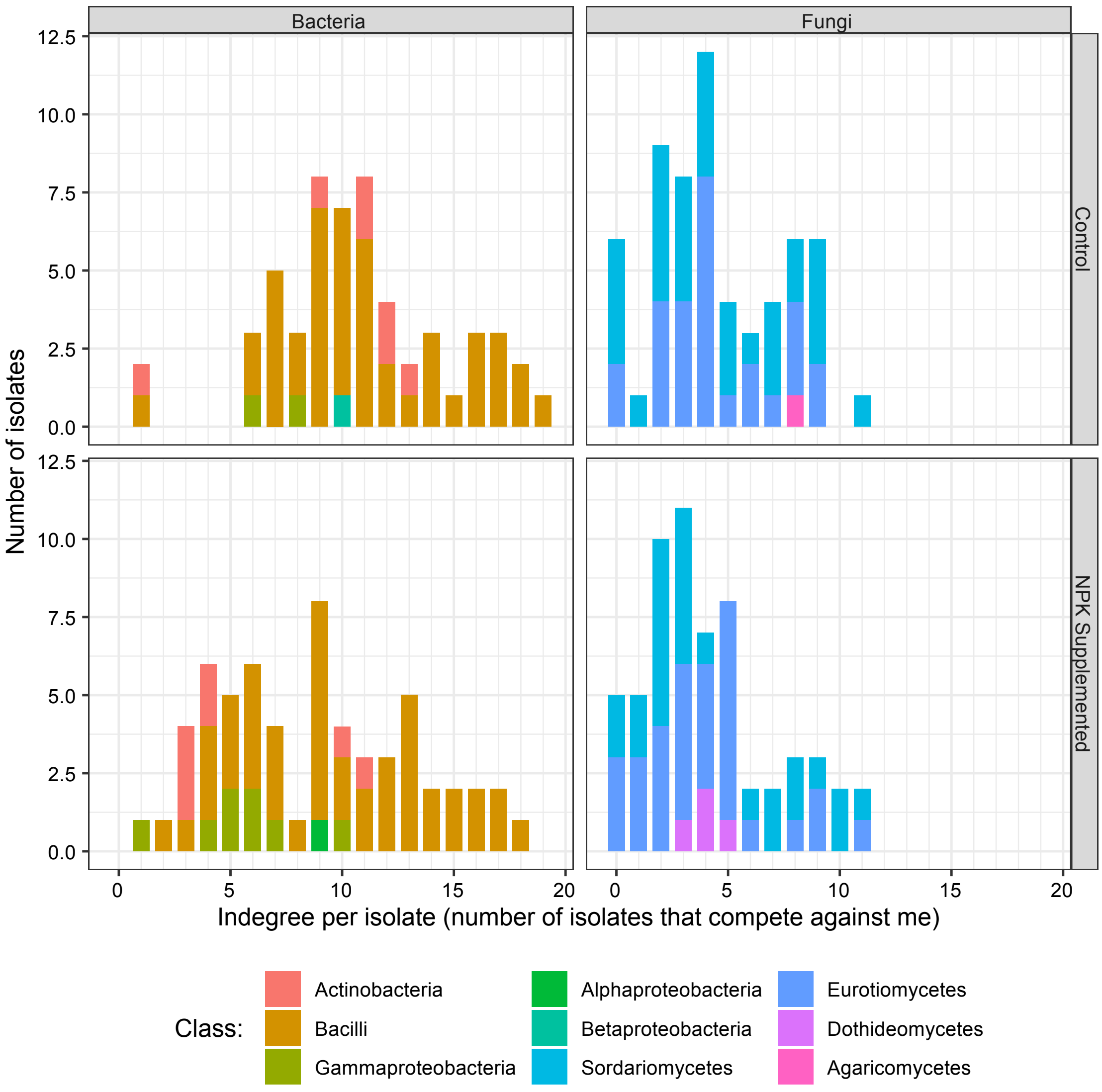

Figure S8: Indegree across treatment (row) and kingdom (column), colored according to the class of the focal isolate.

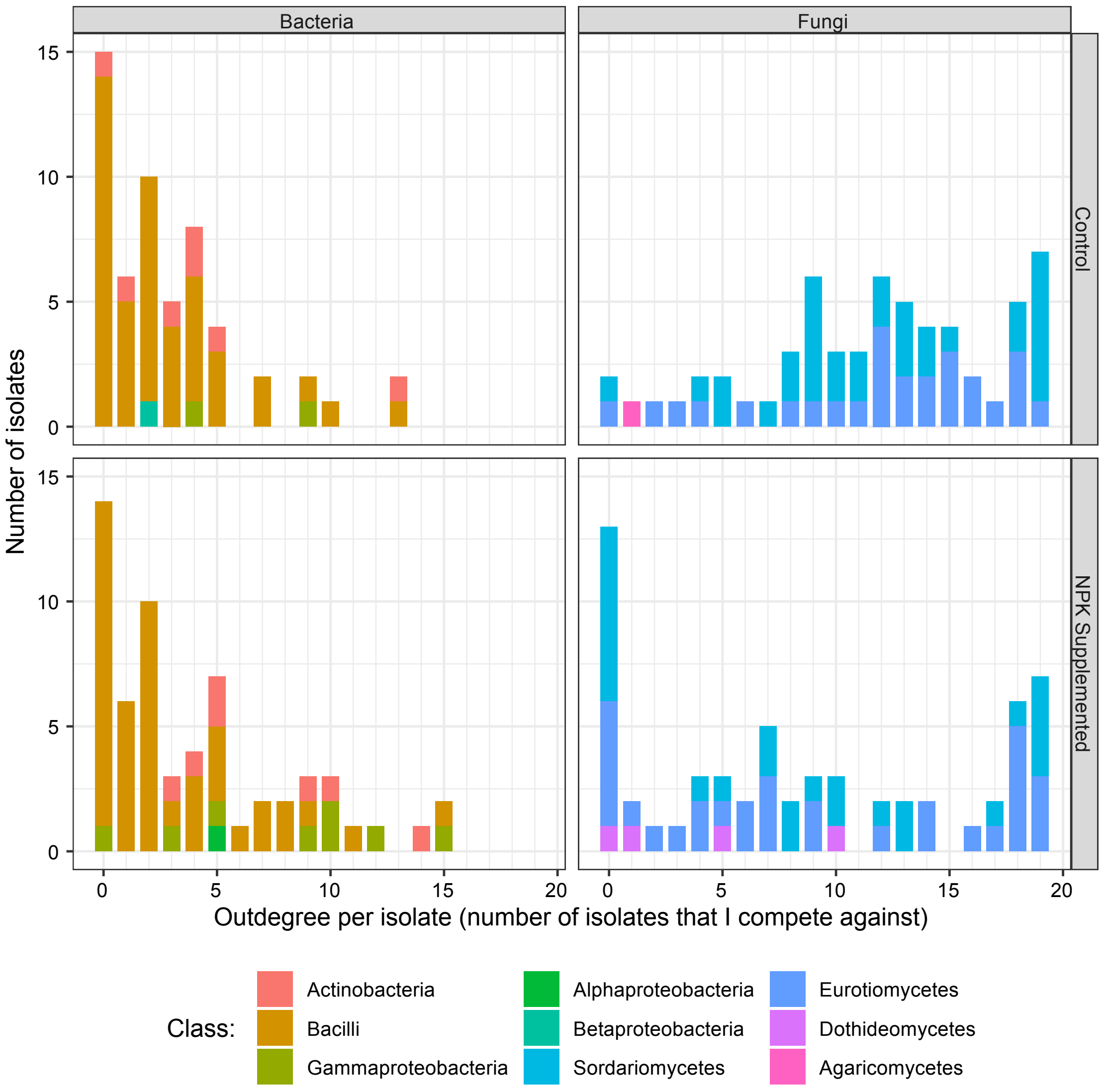

Figure S9: Outdegree across treatment (row) and kingdom (column), colored according to the class of the focal isolate.

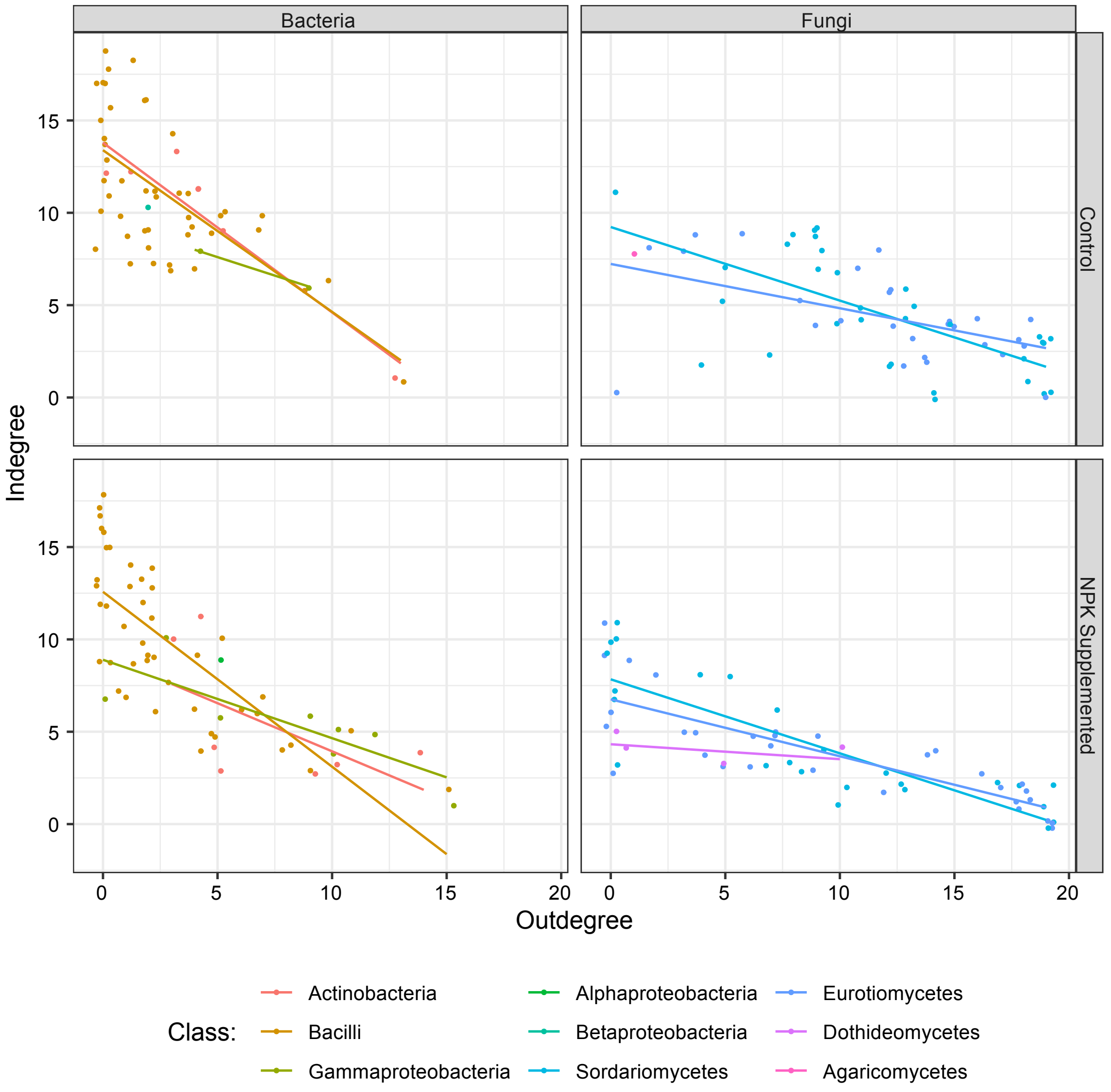

Figure S10: Indegree by outdegree for each isolate (similar to Figure 3), colored according to the class of the focal isolate. Lines indicate linear model fits to isolates within each class. Note that three classes (Alphaproteobacteria, Betaproteobacteria, and Agaricomycetes) were represented by a single isolate each, and thus do not have associated linear models (but the points are still presented).

Table S22: Analysis of variance for indegree vs. outdegree considering the taxonomy of the focal isolate.

Term	df	Sum squared error	Mean squared error	p value
Outdegree	1	2722.54	2722.54	< 0.001
treatment	1	173.49	173.49	< 0.001
kingdom	1	243.40	243.40	< 0.001
phylum	3	54.74	18.25	0.049
class	4	27.12	6.78	0.414
order	7	21.12	3.02	0.876
family	8	92.52	11.56	0.103
Residuals	209	1432.46	6.85	NA
R^2	0.70			

Definitions of network metrics

Local structures (one value for each node/isolate)

Node clustering The proportion of a given nodes neighbors (those nodes directly connected to the focal node) that are also connected to one another.

Degree, also Degree centrality The number of links connected to a given node; can be subset according to link direction. Can be calculated for both binary and weighted networks.

Closeness centrality The average shortest path length from a given node to all other nodes in the network; can be subset according to link direction. Can be calculated for both binary and weighted networks.

Neighbor An informal way to refer to a node that is directly linked to a focal node

Global structures (one value for each network/community)

Network Clustering The proportion of all nodes' neighbors that are also connected to one another. Can also be thought of as the ratio of complete (*i.e.* A interacts with B, B interacts with C, and C interacts with A) to incomplete triangles (*e.g.* A interacts with B and B interacts with C, but A and C do not interact) in the network. Can be calculated for both binary and weighted networks.

Connectance The proportion of all possible links that are realized (*i.e.* the sum of the degree divided by the square the number of nodes in the network).

Mean (inbound/outboud/proportion) degree The average number of links in a given direction each node is connected to. In the case of proportion of degree inbound, the mean of the distribution of the ratio of incoming to outgoing links for each node.

Standard deviation of (inbound/outboud/proportion) degree The square root of the variance of the distribution of inbound/outbound/ratio of in- to outbound links for all nodes.

Skewness of (inbound/outboud/proportion) degree The normalized third moment of the distribution of inbound/outbound/ratio of in- to outbound links for all nodes.

Triad counts For each of the 16 possible combinations of three nodes in a directed network (graphically presented in Table 4), how many of each can be found among the $\binom{n}{3}$ possible three-node subgraphs that can be extracted from a network of size n (*i.e.* having n nodes).

Intransitivity After sorting a network to be as transitive as possible, *i.e.* sorted such that as many links as possible are pointing in the same direction along a linear hierarchy of nodes, intransitive links are those directed back "up" the hierarchy. Intransitivity is the proportion of possible intransitive links that are realized.

Discussion of idiosyncratically significant structural differences

Despite the lack of network-wide evidence for HOI, significant differences in triad abundances between empirical and rewired networks for some leaves suggest potentially idiosyncratic discrepancies for some triads and for HOI within individual communities. Among all 16 possible triads (Table 4), the abundance of only one triad was significantly different between nutrient-amended and control leaves; triad 13 (*i.e.* mutual niche-overlap between a pair of isolates, each of which also compete with a third isolate) was significantly more abundant in control than in NPK-treated leaves. However, this difference was not robust to correction for multiple hypothesis testing (Table 4). More broadly, there were trends in the relative abundance of some triads that were consistent between treatments. In particular, some triads (*e.g.* triads 7, 8, and 10) are rarely present, while others (*e.g.* triad 9) are very abundant, even though all four of these triads involves the same total number of competitive interactions (*i.e.* 3). Similarly, triad 13 is more abundant than other triads having 4 interactions (11, 12, and 14) in endophytic communities from both nutrient-amended and control leaves.

While some communities showed no evidence for HOI, for others one-third or more of total triads were significantly under- or over-represented, and evidence for HOI was more common in networks from NPK-amended than control leaves. Interestingly, there were two triads in which the direction of the discrepancy (over- vs. under-represented in observed vs. rewired communities) differed between NPK-amended and control leaves. Triad 5 (two isolates both significantly overlap the niche of a third) was significantly over-represented in the control network, but under-represented in NPK-amended leaves. In contrast, Triad 9 (as in Triad 5, but where one of the two initial isolates additionally shows significant overlap with the other; see Table 5) was under-represented in the control leaf, but over-representation in two of the NPK-amended leaves. The causes underpinning such idiosyncratic discrepancies are potentially interesting topics for further analyses, and suggest the need for further work exploring the potential for HOI within endophytic communities.

Of note, triads 10 and 14 were never observed in our networks (along with a scarcity of triads 15 and 16). These four triads contain an intransitive cycle in their structure (*i.e.* a Rock-Paper-Scissors relationship; $A \to B \to C \to A$). As noted in the main text, these networks are highly nested and sufficiently high nestedness precludes the possibility of intransitive cycles. As can be noted from Table 5, the majority of these communities are precluded from exhibiting triads 10 and 14 simply by dint of their degree distributions.

Additional comparisons of network structure between treatments

Table S23: Welch's two-sample t-Test comparing z scores calculated from distributions generated by configuration model randomizations of each network. Boxes are colored according to the treatment with the larger value for each metric, ■ for control and ■ for NPK. Empty boxes signify p values > 0.05. The two significant differences have p values of 0.0183 and 0.0153, respectively, when uncorrected for multiple hypothesis testing. Applying such a correction makes all differences non-significant.

Metric	Control	NPK Supplemented	p value
Network Clustering	-0.78	1.52	□
Intransitivity	1.34	0.07	□
1. 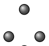	0.44	-1.87	■ (control)
2. 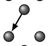	-0.23	2.26	■ (NPK)
3. 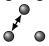	2.00	0.51	□
4. 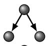	-0.02	-1.99	□
5. 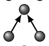	0.61	-1.21	□
6. 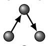	-1.22	-1.67	□
7. 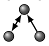	0.38	-0.60	□
8. 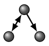	0.77	-0.44	□
9. 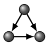	-0.70	0.70	□
12. 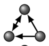	0.65	-0.07	□
13. 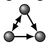	1.34	0.75	□

Table S24: As Table 3, but utilizing metrics calculated on the weighted niche-overlap networks. Welch's two-sample t-Test comparisons of whole-network-scale metrics across treatments. Boxes are colored according to the treatment with the larger value for each metric, ■ for control and ■ for NPK. Color intensity indicates the level of significance, with the one significant difference having a p value of 0.0045. Empty boxes signify p values > 0.05. Network clustering is not defined for weighted networks and is thus not included in this table. p values are uncorrected for multiple hypothesis testing and applying such a correction makes all differences non-significant. We omit the outlying control network with fewer than twenty isolates; inclusion of this community does not change these results qualitatively. Formal definitions of each metric can be found in the following section

Metric	Control	NPK Supplemented	p value
Connectance	0.62	0.60	☐
Intransitivity	0.01	0.01	☐
Mean degree	12.33	12.05	☐
Mean proportion degree inbound	0.50	0.51	☐
Standard deviation of indegree	2.45	2.24	☐
Standard deviation of outdegree	3.29	3.25	☐
Standard deviation of proportion degree inbound	0.12	0.12	☐
Skewness of indegree	-0.17	-0.67	■
Skewness of outdegree	-0.20	-0.18	☐
Skewness of proportion degree inbound	0.19	0.03	☐
